# Innovative Geopolymer Tiles for Indoor Humidity Control: A Comparative Study of Moisture Buffering Performance

**DOI:** 10.1021/acsomega.4c09422

**Published:** 2025-03-03

**Authors:** Gurkan Akarken, Yildiz Yildirim, Ugur Cengiz

**Affiliations:** a Department of Energy Resources and Management, Faculty of Engineering, Çanakkale Onsekiz Mart University, Çanakkale 17010, Türkiye; b AFC Green Technologies R&D, Canakkale Technopark, Sarıcaeli, Çanakkale 17100, Türkiye; c Kale Ceramic R&D Department, Canakkale 17400, Türkiye; d Surface Science Research Laboratory, Department of Chemical Engineering, Faculty of Engineering, Çanakkale Onsekiz Mart University, Çanakkale 17020, Türkiye

## Abstract

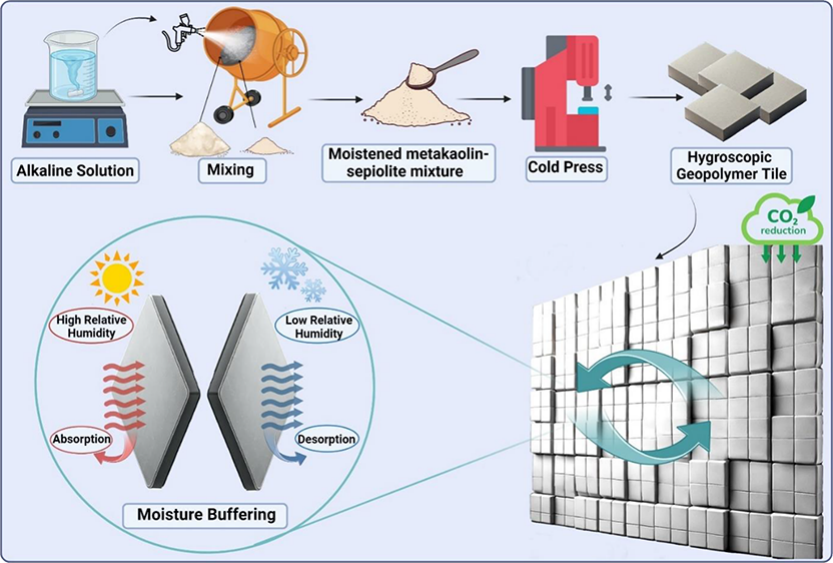

Geopolymers have attracted increasing attention due to their unique properties in the construction industry. In this work, innovative geopolymer tiles were evaluated regarding their potential to control indoor relative humidity as a passive construction material. Our production process systematically develops geopolymer tiles with elevated moisture buffering capabilities using four distinct metakaolins and one commercial metakaolin to make a comparison. A critical metric for evaluating hygroscopic materials’ capacity to control the indoor humidity change is the moisture buffer value (MBV). The geopolymer tiles’ MBV was determined by the Nordtest method in a controlled climate chamber. Additionally, a custom-designed moisture buffer test and strength measurements were conducted, including inspections of the physical appearance after the tiles were submerged in water for 7 days. The results indicate that the geopolymer tiles exhibit exceptional moisture buffering performance, with MBV values ranging between 5.68 and 7.94 (g/m^2^ Δ%HR). These are the highest and one of the first values for geopolymer tile moisture buffer values in the literature so far. The text discusses the advantages and superior performance of these tiles compared with conventional methods, supported by mechanical, morphological, and structural analyses.

## 1 Introduction

Energy demand is increasing rapidly due to global economic development, especially in developing countries. The building industry consumes almost half (45%) of the world’s total energy, with over 40% of that energy was used for heating, ventilation, and air conditioning (HVAC) systems.^1,2^ To mitigate energy consumption in buildings, two primary strategies are employed:^3,4^ one is called an active approach, which involves the efficient use of HVAC systems, and the other one is passive approach, which leverages effective passive materials in construction to reduce energy needs.^5,6^ Researchers have focused on the passive hygrothermal performance of materials that could provide indoor thermal and moisture buffering performance (MBP) not only to conserve energy but also to provide comfortable indoor space in residential environments.^7−10^ Moisture buffering through using hygroscopic building materials can reduce the energy footprint of buildings by up to 30%.^11^ These materials achieve MBP via a porous structure. This kind of structures can absorb excess humidity in indoor spaces. The other way round, they can also desorb the humidity in their pores to the environment when there is insufficient humidity in the living.^12^ Notably, these materials can perform these functions without consuming energy unlike active techniques for controlling indoor relative humidity (RH) that require enormous quantities of energy such as air conditioners, central and portable humidifiers, and ventilators.

Humidity poses various challenges such as accelerating metal corrosion and compromising the integrity of concrete structures by damaging embedded iron components. Besides the negative impacts on the buildings, elevated indoor moisture levels can also negatively impact human comfort and health by promoting condensation on surfaces and facilitating the growth of microorganisms.^13,14^ In the postpandemic world, where individuals spend significant amounts of time indoors, indoor air quality has become a critical factor in human comfort.^15^ Indoor humidity levels that are excessively high in the summer or during periods of high occupancy or too low in the winter can have a negative impact on people’s ability to live and work. Wolkoff reported that low RH contributes to the osmolarity of the upper airways and the stability and physiology of the tear film in the eyes. Many influenza viruses grow and spread more easily under low RH levels.^16^ Thus, maintaining appropriate RH levels in living spaces is crucial for human health and quality of life.

The primary objective of this study was to design and evaluate geopolymer tiles with advanced MBP specifically aimed at stabilizing indoor relative humidity levels to enhance human comfort and health. Unlike conventional materials, which often lack sufficient hygroscopic properties, the novel geopolymer compositions in this research were developed to address humidity-related challenges while maintaining the long-term durability and structural integrity. This work leverages the untapped potential of metakaolin-based geopolymers (GPs) as passive hygroscopic construction materials, providing a sustainable and effective solution for indoor climate control.

The goal of reducing or even eliminating the carbon footprint in the construction sector has become a critical global sustainability objective, as in all industries. GPs are new eco-friendly, sustainable inorganic construction materials derived from activating aluminosilicate feedstock in an alkaline solution. Recently, they have attracted considerable attention^17−24^ due to their mechanical and durability properties, which are comparable to Portland cement, but with significantly lower energy requirements and greenhouse gas emissions during manufacture.^25−27^

The aluminosilicate sources for GPs typically include natural minerals such as metakaolin (MK), volcanic ash, or industrial wastes such as fly ash and slag, reducing production costs and addressing environmental concerns associated with waste disposal. Compared to the traditional Portland cement, GPs are considered green materials due to their reduced CO_2_ emissions in their production.^28−31^ These advantages have spurred interest in various applications, including refractories, construction concrete, biotechnology, antifouling, antigraffiti, weather protection, composites, and heavy metal immobilization.^32−38^ However, there are only a few studies on the MBP of geopolymers for interior applications up to today.^8,39−43^

This research applies the geopolymerization technique to endow indoor wall tiles with MBP, an area where existing studies are sparse. To the best of our knowledge, the geopolymer tiles produced in this study demonstrated the highest moisture buffer values reported in the literature. The research began by producing four distinct metakaolins (MKs) from different commercial kaolins through a high-temperature processing. The geopolymer mortar was then enriched by adjusting the Si, Al, and Na ratios according to the literature.^42^ Cioffi et al. reported the optimization of GP synthesis and determined that the most mesoporous structures formed in the Na/polysialate-siloxo combination with a rate of 83%. Furthermore, the SiO_2_/Al_2_O_3_ ratio must be above 3 to obtain Na/poly sialate-siloxo structures.^44^ Based on these findings, the study used the following molar ratios: SiO_2_/Al_2_O_3_: 4.0, Na_2_O/SiO_2_: 0.35, H_2_O/Na_2_O: 18, and Na_2_O/Al_2_O_3_: 1–1.5.

In this study, the structural and surface properties of the innovative hygroscopic geopolymer tiles were systematically characterized and compared to highlight their unique features. Strength measurements and physical inspections were performed following immersion in water for 7 days to evaluate their durability under extreme conditions. To comprehensively assess the MBP, two advanced methodologies were employed: an adsorption–desorption test conducted in a climate chamber using the Nordtest method to determine the moisture buffer value (MBV) and a custom-designed humidifier system test to simulate real-world indoor conditions. Notably, the hygroscopic geopolymer tiles achieved an unprecedented MBV of 7.94 (g/m^2^ Δ%RH), the highest value reported for geopolymer materials in the literature to date, underscoring their exceptional capability as passive indoor climate regulators.

## 2 Materials and Method

### 2.1 Materials

Four types of kaolins (CC31, K2, CAMP S4, and MBA) were used to produce MK. CC31 kaolin is a product of British W.B.B. K2 kaolin, known as Bulgarian kaolin, is primarily used in vitrified products. CAMP S4 is a vitrified kaolin obtained from the Serina Group. Lastly, MBA kaolin, used for porcelain tiles, and MK750 reference kaolin were acquired from the Imerys Company. The dry strength of these kaolins ranges from 8 to 11 kg/cm^2^, and the firing shrinkage was a maximum of 5% at 1200 °C, although this varies depending on their specific application in ceramics. The performance of the produced MKs was compared to Mefisto L05, a commercial MK sourced from Ceske Lupkove Zavody (Czech Republic). The chemical compositions of the five different kaolins and L05 MK are given in Supporting Information Table S1. Sepiolite, used as a filler in GP tiles, was obtained from Akmin Madencilik in Eskisehir, Turkey. This material is commonly employed in the production of moisture control products for interior wall coatings due to its hygroscopic properties.^45^ The alkaline activator solution was prepared by using water, sodium hydroxide (NaOH), and sodium silicate. The sodium silicate, with a SiO_2_/Na_2_O ratio of 2 and a density of 1.50 g/L, was supplied by Sodel Kimya, Turkey. Sodium hydroxide, with a purity greater than 99.99 wt %, was obtained from Interlab, Istanbul, Turkey.

### 2.2 Metakaolin Preparation

The four different kaolin samples (K2, CC31, MBA, and CAMP S4) were placed in corundum crucibles and heated to 700 °C at 20 °C/min in a Carbolite-Htc 14/30 furnace to obtain the metakaolin (MK) in accordance with the literatuure.^43^ According to the findings of Elimbi et al., 700 °C is the ideal temperature for producing high-quality geopolymers through kaolin calcination.^46^ Once the target temperature (20 °C/min of heating rate) was achieved, it was maintained for 8 h, after which the furnace was allowed to cool down to room temperature. The resulting metakaolin samples stored in a desiccator and micronized using a Retsch Rmo ball mill. The prepared MKs were subsequently readied for application in geopolymer tiles.

### 2.3 Geopolymer Tile Production

**Scheme 1:**
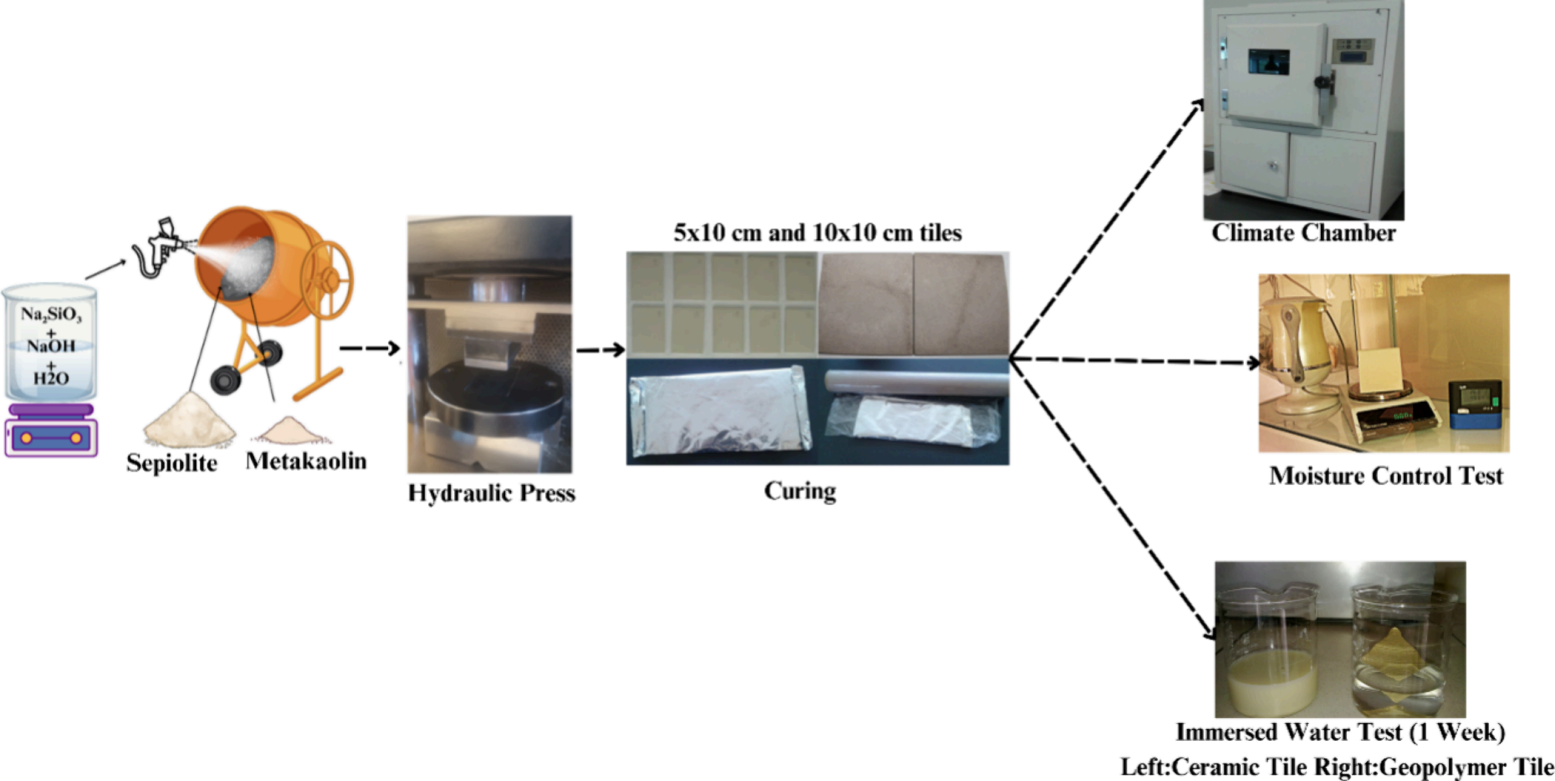
Hygroscopic Geopolymer Tile Production Process and Moisture Buffering Testing Techniques

The composition of the GP tile mixtures is determined according to US Patent 4.349.386. In mineral-based polymers, the specified molar ratios are SiO_2_/Al_2_O_3_: 3.5–4.5, Na_2_O/SiO_2_: 0.20–0.28, and H_2_O/Na_2_O: 15–25 compatible with the literature.^44^ The dry metakaolin and sepiolite were accurately weighed and combined in an Eirich granulator, where they were mixed for 15 min to achieve a homogeneous blend, as illustrated in Scheme 1. The liquid phase was prepared by thoroughly mixing 182 g of sodium silicate, 16.84 g of NaOH, and 136.4 g of water to form a uniform solution. This liquid mixture was then sprayed onto the powder mixture, consisting of 100 g of metakaolin (MK) and 300 g of sepiolite, at a controlled rate of 0.2 L/min, ensuring complete wetting within the granulator. Following the full wetting of the mortar, the mixture was further blended for an additional 5 min, resulting in the formation of geopolymer (GP) mortar. The GP mortar was subsequently pressed using a Nanetti-Mignon hydraulic press at a pressure of 400 kg/cm^2^ to fabricate GP tiles with dimensions of 5 × 10 and 10 × 10 cm. The produced GP tiles were cured at 150 °C for 4 h (Scheme 1). To ensure efficient heat retention within the tiles during the curing phase, test samples were wrapped in aluminum foil and subsequently enclosed in stretch film within a controlled laboratory environment (Scheme 1).

### 2.4 Characterization of Materials

The flexural strength measurements of the GP tiles (5 × 10 and 10 × 10 cm) were performed using the Gabrielli-S.R.L CR5 strength device. The strength values were measured according to the TS EN ISO 10545-4 instruction (Determination of Flexural Strength and Fracture Strength of Ceramic Tiles). The mechanical strength of the GP tiles was measured 1 and 7 days after the reaction’s completion. Additionally, the mechanical strengths were evaluated after the dried tiles were kept in water for 7 days. After being removed from the water, the tiles were dried in an oven at 105 °C. Once cooled to room temperature, their strength measurements were tested.

The chemical compositions of GP tiles were determined by X-ray spectrometry (XRF, Panalytical Axios Max). The mineralogical compositions were obtained by XRD (X’pert pro mpd) and FTIR devices (Bruker Alpha ATR).

Surface morphology was characterized by scanning electron microscopy (SEM-EDS) analysis with a JEOL electron microscope. Surface area changes were measured by using the SSA device. The samples, granulated to a 2 mm grain size from the prepared tiles, were dried in a nitrogen atmosphere, and their measurements were obtained using a Strolein Area Mat II surface area device. The SSA analysis measures the total surface area of a material per unit of mass. This measurement is critical in understanding the material’s capacity for adsorption and reactivity. In the context of geopolymers, a higher SSA generally indicates a greater number of active sites for chemical reactions, which can enhance the material’s MBP.

To determine the particle size distributions, aqueous solutions of kaolin samples were prepared. Hexametaphosphate was chosen as the electrolyte to disperse the particles. For particle size measurements, 100 mL of water and 3 mL of 0.1% hexametaphosphate were added to 10 g of sample. After waiting for 12 h, mechanical mixing was performed for half an hour. The prepared slurry was measured using a Malvern Mastersizer Micro Plus particle size dispersion device, operating with the laser method. The pump speed was set to 2000 rpm, and the measurement time was set to 60 s. Measurements were taken three times, and the average of the analyses was recorded.

#### Moisture Buffering Analysis

Two different tests were performed to evaluate the moisture buffering performance (MBP) of the GP tiles. Currently, there is no established testing protocol for MBP of construction materials. However, various experts in the field have proposed different approaches for its measurement, each involving exposure of the material surface to varying vapor pressure changes. The Nordtest method is one such approach.^47^

This method describes the relationship between moisture release and moisture absorption per surface area as well as the cyclic variation in material relative humidity (RH). The moisture buffer value (MBV) adsorption–desorption performance was determined using an Entek B21 climate chamber device following the Nordtest method. Moisture adsorption–desorption behaviors of the samples were measured during 8 h of adsorption at 75% RH and 16 h of desorption at 33% RH. This procedure was performed 72 h in order the complete three cycles at 23 °C. Five samples of five different geopolymers made from five types of metakaolin were measured in this analysis. MBVs were calculated using the formula provided in the Nordtest project, as shown in eq 1 below:
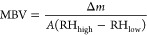
1where MBV is the moisture buffer value in g/(m^2^%RH) and RH_high_ (75%) and RH_low_ (33%) are the relative humidities high and low, respectively. Δ*m* is the mass differential during moisture adsorption and desorption in grams (g), and *A* is the specimen’s open surface area in square meters (m^2^).

Another method for determining the MBP of the hygroscopic tiles was designed for this study. A mechanism with a 1 m^3^ volume glass partition was prepared, as seen in Scheme 1 (humidity control test). A temperature–humidity meter and scales were placed in the glass partition, and the humidity was controlled using boiling KCl (aq) solution. The relative humidity was set to 80%. The change in the weight of the tiles was measured and reported as moisture differences in tiles’ structures.

## 3 Results and Discussion

### 3.1 Production of Geopolymer Tiles

To produce hygroscopic GP tiles, it is essential to first transform kaolin into metakaolin under optimal conditions (heating rate and calcination time). A calcination temperature of 700 °C was selected as the most appropriate for this conversion, based on the established literature recommendations.^43^ The dehydroxylation of kaolin begins between 450 and 500 °C, influenced by factors such as crystal size, crystallinity, and heating rate. The literature results showed that the peak temperatures for all kaolins were ranged from 500 to 550 °C, with complete removal of OH groups occurring between 650 and 680 °C.^43^ Consequently, 700 °C was determined as the minimum temperature for producing metakaolin, aligning with the existing literature. Kamseu et al. confirmed that 700 °C is optimal for achieving high-quality GPs in their study on kaolin calcination.^43^ MK750 kaolin was chosen as the reference kaolin, and it underwent calcination at varying durations and heating rates, with subsequent analysis of the changes in specific surface area (SSA) (refer to Supporting Information Table S2). The objective of the experiment was to determine the conditions under which MK750 achieves the SSA value closest to the initial value of 13.10 m^2^/g before thermal exposure. A minimal structural change after calcination at 700 °C suggests an amorphous state. Examination of Table S2 in the Supporting Information reveals that SSA values of 13.5 and 13.75 m^2^/g were achieved with heating rates of 20 °C/min for 8 h and 50 °C/min for 10 h, respectively. Given the cost-effectiveness and proximity to the standard value, the optimal conversion conditions for kaolin to metakaolin were established as 700 °C, with a heating rate of 20 °C/min and an exposure time of 8 h.

The production of hygroscopic GP tiles was optimized by adjusting the press mechanism and curing temperature. Commercially available L05 metakaolin-sepiolite powder, after being moistened with an alkaline solution, was pressed into tiles measuring 5 × 10 cm under pressures ranging from 250 to 400 kg/cm^2^. The strength of the tiles was evaluated as a function of the applied press pressure (Supporting Information Table S3). The visual appearance of the GP tiles was also assessed to determine the optimal press pressure, with the pressure that resulted in noncracking tiles being chosen as the ideal working condition. An increase in hydraulic press pressure found a correlation with higher strength values. The optimal results, which included both enhanced strength and noncracking tiles, were achieved at a press pressure of 400 kg/cm^2^. Therefore, a pressure of 400 kg/cm^2^ was selected for the production of GP tiles in this study.

The effects of the curing temperature and time on the GP tiles were also investigated in relation to strength values. Three different curing temperatures (80, 100, and 150 °C) were applied, and the strength values were measured after the GP tiles were allowed to cool to room temperature (Table 1).

**Table 1:** Curing Temperature–Strength Change

**temperature (°C)**	**time (h)**	**strength(kg/cm**^**2**^^a^**)**
80	4	30.9
	6	47.8
	8	62.3
	10	90.2
100	4	34.5
	6	49.8
	8	115.6
	10	125.8
150	4	72.9
	6	132.8
	8	147.9
	10	183.6

a Average of three 5 × 10 cm tablets.

The optimum curing temperature was determined to be 150 °C for 4 h, as this condition yielded both improved mechanical strength values and a noncracking tile appearance (Table 1). The experiments on curing temperature and pressure demonstrated that the best tile formation was achieved with a press pressure of 400 kg/cm^2^ and a curing temperature of 150 °C for 4 h. These parameters were subsequently applied to all of the GP tiles in this study.

### 3.2 Effect of Different MKs to Phase Compositions and Structure Characterizations

**Figure 1:**
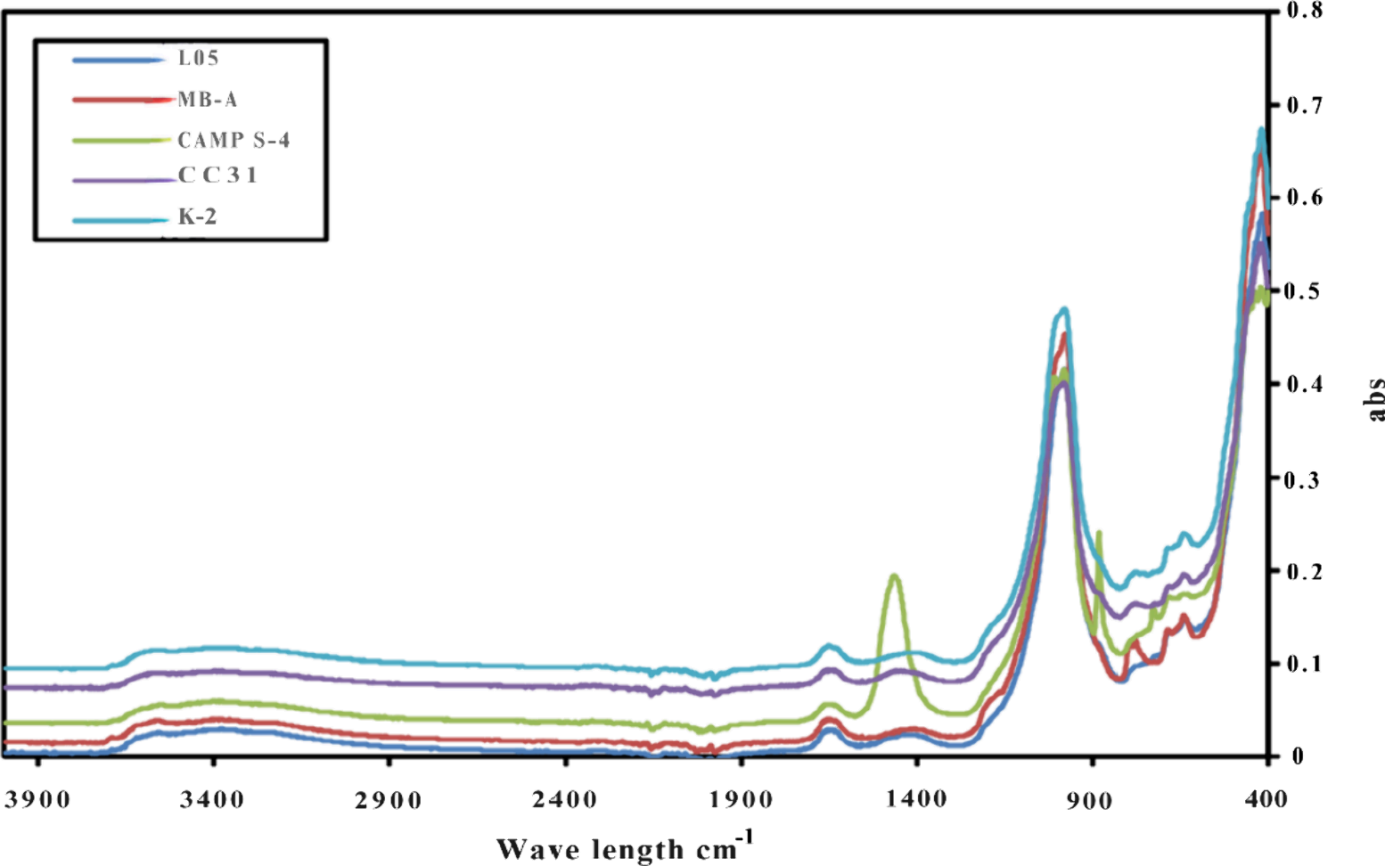
FTIR spectrum comparison of geopolymers produced from five different types of metakaolin.

Two different sizes of hygroscopic GP tiles were produced: 5 × 10 and 10 × 10 cm, both at a press pressure of 400 kg/cm^2^ and a curing temperature of 150 °C for 4 h. The FTIR spectrum samples were obtained from the 5 × 10 cm tiles, and the spectra of the different GP tiles are presented in Figure 1.

The geopolymers prepared from L05 metakaolin, selected as the reference metakaolin in this study, along with K-2, CC31, MK-A, and CAMP S-4 metakaolins, exhibit a hydrosodalite/Na-PSS structure. The broad peak in the 3600–3000 cm^–1^ band in the FTIR spectra is attributed to the O–H bonds in the hydrosodalite structure.^48^ Although not very prominent, the narrow absorption band observed at 3650 cm^–1^ indicates the presence of O–H bonds surrounded by O and Na atoms. This band is particularly evident in the geopolymer obtained from K-2 metakaolin. The 1640–1650 cm^–1^ range corresponds to the stress peak of the O–H within the crystal. The 1430–1440 cm^–1^ band is associated with the Na–O bond, which is located on the outer atoms of the formed structure and possesses a high ion exchange capacity. The broad peak at 979 cm^–1^ represents the Si–O and Al–O asymmetric atomic vibrations in Si or Al-centered [(Si, Al)O_4_] tetrahedrals.^49^ The bands at 730–662 cm^–1^ are due to symmetric atomic vibrations,^50,51^ while the bands at 458–425 cm^–1^ are indicative of Si(Al)–O deformation vibrations.

All GP tiles’ FTIR spectra are consistent, except for Camp S4. The FTIR spectrum of the metakaolin forms is also provided in Supporting Information Figure S1. When the geopolymer structure formed is evaluated in the comparison chart in Figure 1, it is evident that the ion exchange capacity of the geopolymer formed with Camp S4 is high. Therefore, equal amounts of glass water, NaOH, and sepiolite were used for all structures in the study. Since the amount of MK formed in Camp S4 was less than in the others, the alkaline materials used in the recipes reacted with sepiolite. Consequently, Si–O bending vibrations (820 cm^–1^) were strong in the geopolymer prepared with MB-A.^52^ Si–O bending vibrations were also observed in the K2 and CC31. Similarly, Al–O–Si bending vibrations increased in MB-A, K2, and CC31, respectively. This indicates an increase in the intercrystalline stress.

The XRD patterns of five different GP tiles are given in Supporting Information Figures S2–S6. When the patterns were evaluated qualitatively, it is evident that sepiolite, quartz, and the amorphous phase are the main components. Calcite from sepiolite and mica from metakaolin are present as trace minerals. The peak of the amorphous phase is observed at 27° 2θ. The areas of the crystalline and amorphous phases were determined, and the percentages of amorphous and crystalline contents were calculated using the XRD graphs (Table 2). The amorphous structure originates from the geopolymer matrix, while the crystalline phase is attributed to sepiolite, quartz, mica, and impurity minerals.

**Table 2:** XRD Phase Analyses of GP Tiles

	**crystal area**	**amorph area**		
GP tiles	**(unit)**^**2**^	**(unit)**^**2**^	**amorph** phase %	**crystal** phase %
L05	1247.0	2026.6	61.91	38.09
K2	1816.6	2342.6	56.32	43.68
CC 31	1789.7	2129.1	54.33	45.67
MBA	2705.1	2483.2	47.86	52.14
CAMP S4	2023.7	1199.5	37.21	62.79

As shown in Table 2, the crystalline phase increases as the amorphous phase decreases. In this case, apart from the sepiolite used as a filler material, the glass water required for the geopolymer mechanism remains as a residue in the NaOH environment. These alkaline materials are likely to produce different crystallizations. Microstructural analyses were performed to detect the resulting structures. EDS analysis performed in conjunction with SEM and provided detailed information about the formed structures.

Evaluation of the morphology and microstructure of the produced tiles was done with SEM and EDS analyses. Detailed SEM and EDS images of the tiles in different scales are given in Supporting Information Figures S7–S16. In addition, some of the images are shown in Table 3.

**Table 3:**
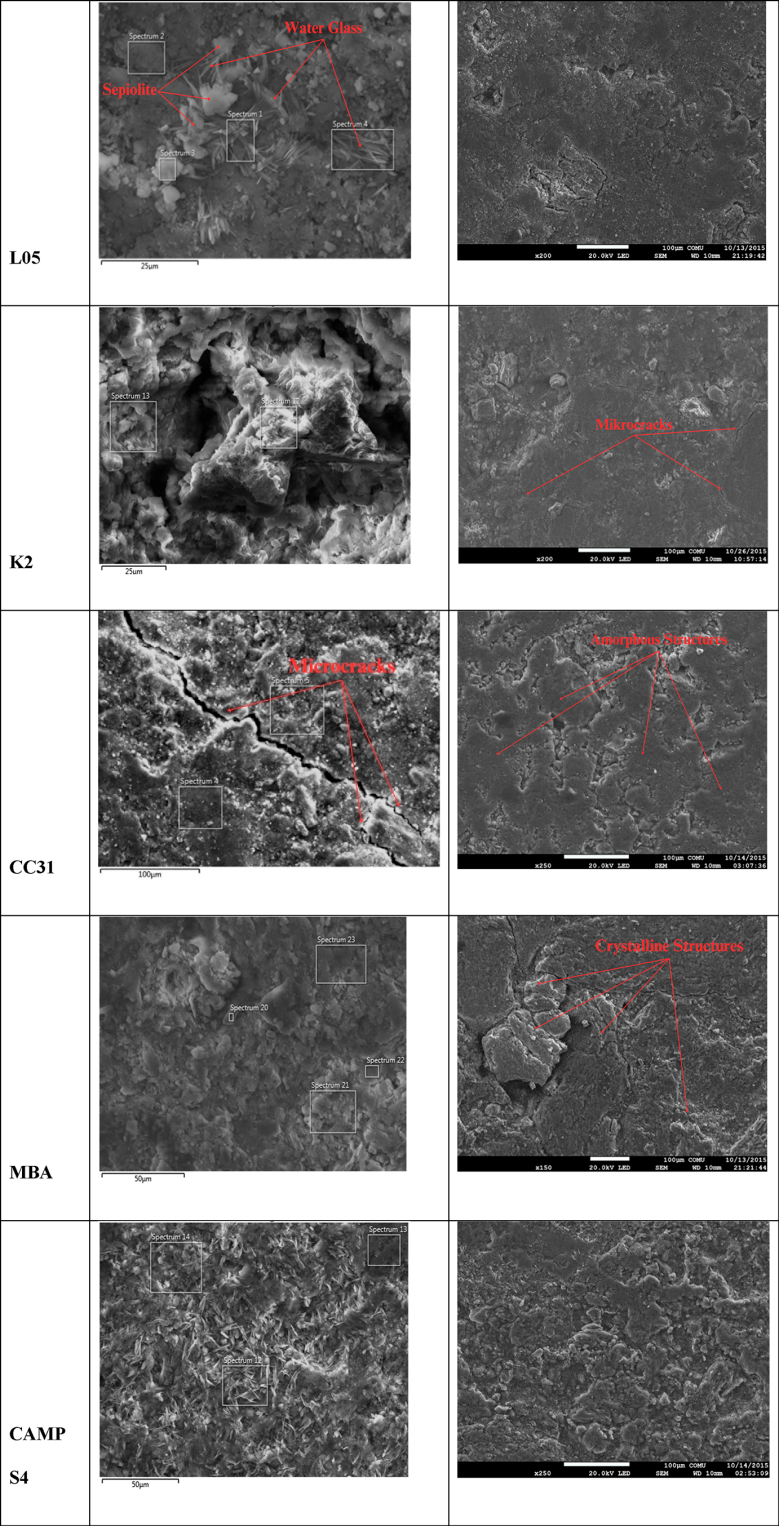
SEM Micrographs of Geopolymer Tiles

L05 GP tile’s SEM images show that the amorphous geopolymer structure was homogeneously dispersed. In microstructural examination, the white-edged structures varying between 2 and 5 μ are calcite crystals accompanying sepiolite. Tubes can be interpreted as sepiolite crystals, needle-like structures as water glass crystals attached to unreacted sepiolite, and sharp-edged grains as quartz crystals. Although it is seen that microcracks occur in the structure, they are not very common.

When the SEM and EDS images of geopolymer tiles prepared with K-2 kaolin are examined, it is observed that the amorphous geopolymer structure is spread homogeneously. Quartz grains larger than 5 μm in size were found within the amorphous structure. Sepiolite crystals are also homogeneously distributed, and the sizes of mica minerals are below 5 μm. The distribution of microcracks in the structure is low, although their size can exceed 100 μm. In the geopolymer matrix, the Si/Al ratio varies between 2.8 and 3.5.

In the case of CC31 geopolymer tiles, gaps between 2 and 5 μm were formed and the occurrence of microcracks increased. The geopolymer amorphous structures were observed to be in the form of sheets. The Si/Al ratio ranged from 2.5 to 3.0. Although there was an increase in the number of quartz grains, their sizes remained below 5 μm. Considering the shape and transparency of mica grains, the structure can be identified as muscovite.

In the analysis of MBA tiles, it was seen that the amorphous structure decreased, and the crystalline phases increased. It was observed that quartz and sepiolite crystals became larger than 50 μm due to agglomeration. Microcracks were observed locally. Gaps have been observed, even though they are not very common. The Si/Al ratio was between 2.75 and 3.2.

Lastly, in the micrographs of CAMP S4, the amorphous structure quantitatively decreased and crystalline phases became more prominent. Another notable change was the increase in needle-like structures containing NaSiO_3_. Quartz grains were prominent. In the geopolymer amorphous areas, the Si/Al ratio ranged from 2.5 to 2.75.

Two different analyses were performed to understand and interpret the correlation between particle size and MBP of the hygroscopic geopolymer tiles: specific surface area (SSA) analysis and particle size distribution analysis. The SSA results for the geopolymer (GP) tiles are summarized in Table 4. The SSA of geopolymer tiles varies due to the presence of nanosized pores in their structures. The results indicate that the L05 GP tile had the most hydrosodalite structure.

**Table 4:** Specific Surface Area (SSA) Results of GP Tiles

Surface	**L05**	**K2**	**CC31**	**MBA**	**CAMP S4**
Area (m^2^/g)	7.45	5.57	5.35	4.230	4.080

It has been demonstrated in several investigations carried out in climate chambers that the interior structure, particularly the distribution of particle sizes, had a greater impact on the absorption of moisture.^53−55^ Average particle size diameters of the MK’s are (in μm) L05:3.00, K-2:3.09, CC 3:6.63, MB-A: 5.90, and CAMP S-4:10.74. Particle size distribution analysis is also provided in Supporting Information Figure S17. Among the tested samples, L05 exhibited the smallest grain size, while the cumulative analysis curves indicated that CAMP S4 had the coarsest structure. MBA and CC31 had nearly identical grain sizes.

Generally, smaller grain sizes lead to a higher SSA because smaller particles have a greater surface area, relative to their volume. According to the particle size and SSA results, it was the same for this study. The geopolymer tile with the highest SSA value (L05) also demonstrated the highest smallest particle size and vice versa as well. Similarly, the SSA values and MBVs of the other geopolymer tiles were found to be directly proportional.

According to Kamseu et al. (2018), pore structure is an important factor that impacts how alkali-activated building materials interact with moisture.^43^ The higher SSA value means more nanomicroparticles per unit and more pores at the same time. All the SSA results given in Table 4 are in accordance with the MBV results given in Table 6. The highest SSA value belongs to the L05 tile, along with its MBP and MBV. To sum up, the analyses of MBP and MBV showed remarkable results compared to other published studies.

**Table 5:** Flexural Strength Changes in Tiles Immersed in Water

	**30 days strength** (kg/cm^2^)^a^	
**tile**	**before water**	**after water**
L05	190.9 ± 0.1	181.4 ± 0.4
K2	168.9 ± 0.1	165.5 ± 0.2
CC31	138.7 ± 0.4	130.4 ± 0.3
MBA	122.4 ± 0.2	116.3 ± 0.4
CAMP S4	83.3 ± 0.3	75.0 ± 0.1

a The average of five 5 × 10 cm tablets was used to determine the strength.

**Table 6:** Comparison of Moisture Buffer Values (MBV) of This Study and Previous Published Papers

**study**	**product**	**highest MBVg/(m**^**2**^**%RH)**	**Nordtest categorization**
(7)	bilayered alkali-activated material	2.71	Excellent
(56)	hemp-clay composites	2.33	Excellent
(57)	wood-cement composite	1.16	Good
(58)	lime cement plasters	1.36	Good
(59)	date palm cement	3.79	Excellent
(60)	cement mortar with porogenic additives	2.6	Excellent
(61)	recycled concrete	0.88	Moderate
(62)	hemp concrete	2.15	Excellent
(63)	hemp-lime concrete	2.02	Excellent
(63)	flax lime concrete	2.27	Excellent
(54)	unfired clay masonry	3.73	Excellent
(64)	perlite	0,23	Limited
(8)	MK-based geopolymer: Si/Al = 3.0	5.62	Excellent
(8)	MK-based geopolymer: Si/Al = 2.6	4.16	Excellent
(8)	MK-based geopolymer: Si/Al = 2.2	5.80	Excellent
(8)	MK-based geopolymer: Si/Al = 1.8	6.81	Excellent
(64)	cellular concrete	0.74	Moderate
(39)	fly ash-based geopolymer	5.61	Excellent
(40)	MK-based geopolymer	5.20	Excellent
(41)	cork-added fly ash geopolymer	1.89	Good
(42)	MK-based geopolymer: Si/Al = 3	5.65	Excellent
this study	L05 GP tile: Si/Al = 3–3.9	7.94	Excellent
this study	K2 GP tile: Si/Al = 2.8–3.5	7.46	Excellent
this study	CC31 GP tile: Si/Al = 2.5–3	7.67	Excellent
this study	MBA GP tile: Si/Al = 2.75–3.2	6.90	Excellent
this study	CAMPS S4 GP tile: Si/Al = 2.5–2.75	5.68	Excellent

The relationship between particle size, SSA, and moisture buffer value (MBV) suggests that the microstructural characteristics of the geopolymer tiles significantly impact their performance. The increased surface area provided by smaller particle sizes enhances the material’s ability to interact with moisture, improving its buffering capacity. This finding underscores the importance of controlling the particle size and porosity in the development of advanced geopolymers for specific applications requiring moisture management. He et al. also provided this justification for the direct relationship between particle size, SSA, and MBV.^12^

### 3.3 Strength Measurement Results

**Figure 2:**
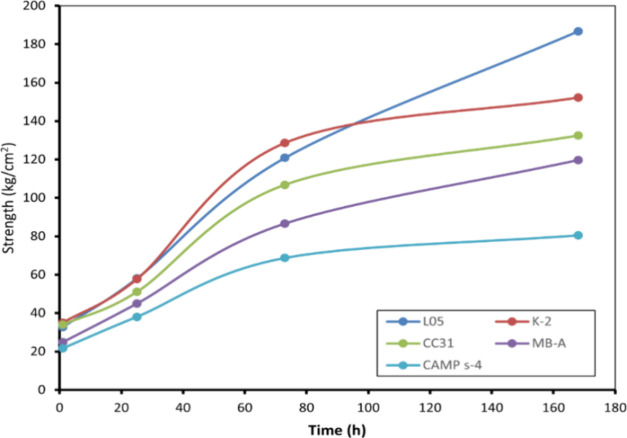
Flexural strength values of geopolymer tiles against time (h).

Flexural strength measurements were conducted using three point bending device on 5 × 10 cm geopolymer tiles prepared with different types of MK. These measurements were taken at 1 h, 1 day, 3 days, and 7 days after the curing period in the oven. The results are presented in Figure 2. The determined strength values illustrate that the increase in strength for L05 metakaolin and MBA tiles was more rapid than that for the others. This phenomenon can be attributed to the continued geopolymerization in L05 and MBA metakaolins, whereas the reaction rate slowed in tiles made with other MKs.

Although the SiO_2_/Al_2_O_3_ ratio was prepared as 4 in the samples with different MKs, the Si/Al ratios in the geopolymer part of the SEM-EDS analyses revealed that SiO_2_/Al_2_O_3_ was 3.90 in the L05 geopolymer tile, while it decreased to around 3 in tiles prepared with MBA and CAMP S4. This shift indicates a transition from the hydrosodalite and Na/poli-sialate-siloxo structure to the nepheline or Na-PS structure, which corresponds to a decrease in strength.

Strength tests were conducted on samples that had been immersed in water for 7 days, as illustrated in Scheme 1 (immersed water test). After their removal from the water, the tiles were subjected to a drying process in an oven at 105 °C followed by cooling to room temperature, and then their strength values were assessed. The results, detailed in Table 4, reveal a significant difference in water resistance between the raw ceramic tile and the geopolymer (GP) tile. While the raw ceramic tile dissolved and dispersed completely after immersion, the GP tile showed no signs of physical degradation, maintaining its original appearance. This observation highlights the superior durability of GP tiles compared to traditional ceramics upon exposure to water.

The inherent durability of the hygroscopic GP tiles can be attributed to the geopolymerization process, a condensation mechanism that facilitates rapid strength development and enhances the structural integrity of the tiles. As demonstrated in Scheme 1 (immersed water test), the raw ceramic tile readily disintegrated in water, whereas the geopolymer tile remained intact, further substantiating the water-resistant properties of the GP material. The strength test results corroborate these findings, indicating that GP tiles possess a notable resistance to water-induced damage.

The observed reduction in strength values, ranging from 3 to 10%, may be explained by the penetration of water into microcracks within the tile structure, where it likely reacts with any unreacted glassy phase. Additionally, the capillary spaces on the surface of the tiles could have absorbed water, which subsequently entered the tiles through these capillary cracks, leading to a minor reduction in the overall strength. Despite this slight decrease, the results affirm the resilience of GP tiles in maintaining structural integrity under prolonged water exposure, making them a promising alternative to conventional ceramic materials, particularly in environments prone to moisture.

### 3.4 Determination of Moisture Buffering Performance (MBP)

The moisture buffering performance (MBP) of a material is a critical indicator of its ability to exchange moisture through its surface when subjected to cyclic variations in relative humidity (RH).^40^ In this study, the adsorption–desorption performance of geopolymer (GP) tiles was assessed by using a controlled climate chamber. Over a 24 h period, the moisture adsorption–desorption behavior was meticulously monitored, with 8 h dedicated to adsorption at 75% RH and 16 h to desorption at 33% RH, following the standardized Nordtest method. For this analysis, five specimens of each metakaolin (MK) tile type were examined over three consecutive cycles. Detailed data from these climate chamber cycles are provided in Supporting Information Tables S5–S9.

**Figure 3:**
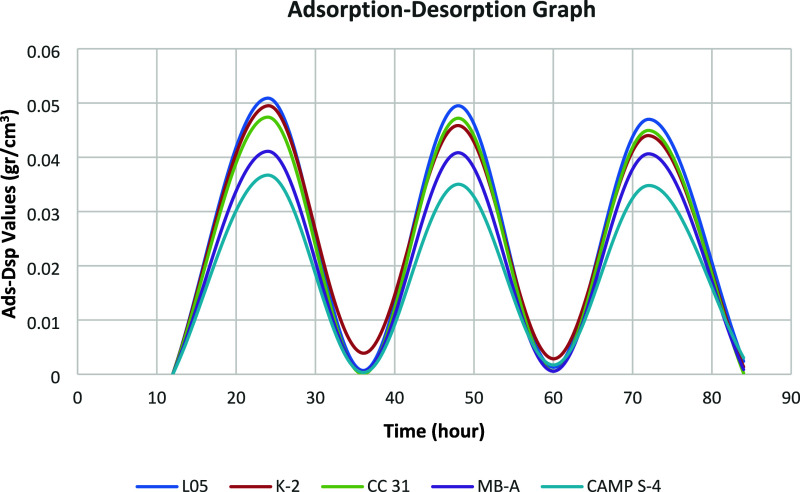
Adsorption–desorption graph of geopolymers prepared with different metakaolins.

The moisture buffering value (MBV) of the hygroscopic GP tiles, a quantitative measure of their moisture management capability, was calculated using the specific equation outlined in Section 2.4. This calculation utilized the average values derived from the five specimens per GP tile type over three cycles, ensuring robust and reliable results. The MBV outcomes were then compared with those documented in prior studies, offering a benchmark for evaluating the performance of these GP tiles. Additionally, a comparative analysis of the adsorption–desorption values for the GP tiles is illustrated in Figure 3, providing a visual representation of their efficiency in moisture regulation.

This comprehensive analysis underscores the efficacy of GP tiles in moisture management, highlighting their potential for applications in which precise moisture control is essential. The MBP characteristics demonstrated by these tiles suggest that they could play a significant role in enhancing indoor air quality and overall comfort in buildings, particularly in environments where fluctuations in humidity are common. The findings from this study position GP tiles as a viable and effective solution for moisture regulation in various architectural and construction contexts.

The data presented in Figure 3 illustrate that the adsorption–desorption values for the geopolymer tiles varied between 0.030 and 0.052 g/cm^3^. This range indicates a robust capacity for moisture management within the tiles. The close consistency of these values across multiple cycles underscores the material’s ability to efficiently release the moisture it absorbs, with minimal variation in performance. This repetitive stability is indicative of the tiles’ ability to desorb nearly the entire amount of moisture absorbed during the high-humidity phase of each cycle.

Moreover, when the relative humidity was lowered, the hygroscopic geopolymer tiles demonstrated the ability to return 95–98% of the absorbed moisture back into the environment while maintaining their moisture absorption capacity in subsequent cycles. This suggests that the material not only absorbs moisture effectively but also releases it in a controlled manner, which is crucial for maintaining indoor air quality and preventing issues related to excess humidity.

**Figure 4:**
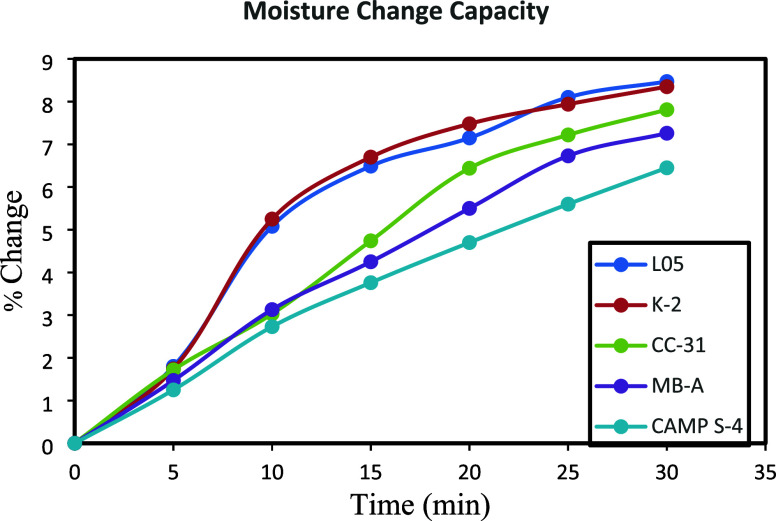
Geopolymer tile moisture change capacity graph.

The setup in Scheme 1 (moisture control test) was prepared for another determination of MBP. In this process, 10 × 10 cm GP tiles were used. Water with KCl solution was boiled to provide humidity. The relative humidity of the environment was increased up to 80%. By keeping this relative humidity constant, the change in the weight of the tile as it absorbed the humidity in the environment was graphed by reading it from the moisture meter (Figure 4).

As the amount of nanoparticles formed in hygroscopic geopolymer tiles increases, the MBP also increases. The MBP of the L05 and K2 geopolymer tiles shows the same performance. The amount of moisture buffer for CAMP S4 is low, but it changes linearly over time.

Table 6 represents the MBV of GP tiles and compares them to several building materials. Although there are just a few published papers about the moisture buffering behaviors of GP tiles, a comparison has been made with various kinds of building materials. The MBV of the building materials is classified through the Nordtest method (Excellent, MBV > 2; Good, 1 < MBV < 2; Moderate, 0.5 < MBV < 1; Limited, 0.2 < MBV < 0.5; Negligible, MBV < 0.2).^43^ Considering all the values obtained in this study, excellent MBVs were achieved. GP tiles have values that are better than those in previous publications (Table 6).

As shown in Table 5, the MBV of the GP tiles in this study are among the highest reported in the literature, with values reaching up to 7.94 g/(m^2^%RH). These values categorize the tiles as Excellent according to the Nordtest classification, surpassing many other building materials, including hemp-clay composites, bilayered alkali-activated materials, and even recycled concrete. This superior MBP indicates that the GP tiles have significant potential to effectively regulate indoor humidity, which is a crucial factor for indoor comfort and health.

Comparing the MBV values from this study with those of other materials presented in Table 5 clearly demonstrates the outstanding performance of the GP tiles. For instance, materials such as lime-cement plasters (MBV = 1.36) and wood-cement composites (MBV = 1.16), which fall into the Good category, perform significantly less effectively than the GP tiles. Even some advanced materials like bilayered alkali-activated materials (MBV = 2.71) and hemp-lime concrete (MBV = 2.02), while classified as Excellent, show lower MBP compared to the GP tiles in this study. These values highlight a distinctive advantage of the GP tiles, which perform better than existing moisture-regulating materials, suggesting their potential to become an industry-leading solution for passive humidity control in buildings.

Notably, the data presented in Table 5 indicate that there is no simple correlation between the Si/Al ratio and the MBV. While it might be expected that an increase in the Si/Al ratio would lead to a higher MBP, due to the larger proportion of gel pores, which theoretically enhances moisture absorption capacity, the relationship is more complex. To fully understand the moisture behavior of a material, it is crucial to consider factors beyond the Si/Al ratio. Parameters such as water vapor permeability, the number of accessible and interconnected gel pores, hydrophilicity, and pore volume distribution also play significant roles.^8^ These factors collectively influence how a material interacts with moisture, thereby affecting its overall MBP. A comprehensive analysis that incorporates these variables is necessary to gain a deeper understanding of the moisture absorption and desorption dynamics in geopolymer materials.

In real-world applications, moisture buffering materials play a vital role in enhancing indoor air quality, particularly in buildings experiencing fluctuating humidity levels. The GP tiles’ high moisture buffering capacity makes them suitable for buildings in regions with high humidity variation, as they can absorb excess moisture during periods of high humidity and release it during drier conditions. This ability to regulate indoor moisture levels passively can significantly contribute to energy savings by reducing the need for mechanical ventilation systems or air conditioning, both of which consume substantial amounts of energy. By mitigating moisture fluctuations, GP tiles can help maintain a more stable and comfortable indoor climate.

Furthermore, the high MBP of the GP tiles has important implications for building durability. Traditional materials, such as cement-based products, are vulnerable to moisture-related issues, such as mold growth and material degradation over time. In contrast, the GP tiles can help prevent these problems by maintaining a stable indoor humidity level, thereby enhancing the longevity of the building. This aspect is particularly valuable in areas where buildings are exposed to high humidity, which often accelerates the degradation of materials such as wood or cement.

While materials like hemp-lime concrete and bilayered alkali-activated materials offer promising moisture buffering capabilities, they are limited by factors such as material degradation or susceptibility to damage under sustained moisture exposure. The GP tiles, however, offer a more resilient and durable solution, making them ideal for applications requiring long-term moisture regulation. Their exceptional durability combined with high moisture buffering capacity makes them a more reliable option for high-performance buildings.

The performance of GP tiles compared to other materials presented in Table 5 shows a clear advantage in moisture buffering, durability, and sustainability. The findings from this study confirm that geopolymer tiles are not only a novel material for managing indoor humidity but also present a viable and sustainable alternative to conventional building materials. By offering an efficient solution for moisture regulation, the tiles contribute to improving indoor environmental quality, energy efficiency, and building longevity.

Additionally, the low environmental impact of geopolymer materials due to their production from abundant waste material positions them as an essential component in the development of sustainable building practices. The low carbon footprint associated with geopolymer production is a key advantage at a time when the construction industry is increasingly focused on reducing its environmental impact. By replacement of more traditional materials with geopolymer tiles, significant contributions can be made to reducing building-related carbon emissions.

## 4 Conclusions

This study aimed to determine the optimum conditions for producing metakaolin from kaolins with different properties, followed by the preparation and evaluation of geopolymer (GP) tiles. Initially, recipes with the same stoichiometric ratio were prepared for all of the metakaolins to produce hygroscopic GP tiles. In the second stage, the optimum pressure for the hydraulic press was determined to achieve the best hygroscopic GP tile production.

1. Structural analyses including strength, FTIR, SEM, XRD, and surface characterizations such as SEM, EDS, and SSA were performed on the produced tiles. The SEM and EDS analyses revealed that the amorphous geopolymer structure was spread homogeneously with variations in quartz grain sizes, mica mineral sizes, and the distribution of microcracks among different GP tiles. For instance, the K-2 geopolymer tiles exhibited low microcrack distribution but larger microcrack sizes, while CC31 tiles showed increased microcracks and gaps between 2 and 5 μm.
2. Strength measurements conducted on 5 × 10 cm geopolymer tiles at various intervals after curing showed that the strength increase for L05 and MBA tiles was more rapid compared to others. This was attributed to continued geopolymerization in these metakaolins. Although the SiO_2_/Al_2_O_3_ ratio was initially set at 4 for all samples, SEM-EDS analyses revealed a decrease in this ratio for MBA and CAMP S4 tiles, indicating a structural shift that corresponded with a decrease in strength.
3. Hygroscopic geopolymer tiles were also tested for durability by immersing them in water for 7 days. The results showed that while raw ceramic tiles completely dispersed, GP tiles remained intact, demonstrating superior water resistance and durability. The strength of GP tiles decreased by 3–10% after water exposure, likely due to water entering through microcracks and reacting with unreacted glass water or filling capillary spaces.
4. Particle size distribution tests and specific surface area (SSA) analyses indicated a direct correlation between particle size and MBP. L05 metakaolin had the smallest grain size, whereas CAMP S4 had the coarsest structure. The L05 GP tile had the highest SSA and MBV, showing a direct proportionality between SSA values and MBVs among the tested GP tiles.
5. The MBP of the tiles was tested using two methods: the adsorption–desorption test through the Nordtest method and a custom-made humidifier system. Detailed climate chamber cycle data indicated that the adsorption–desorption values ranged between 0.030 and 0.052 g/cm^3^. These values remained consistent over repeated cycles, indicating stable performance. The pore structure, specifically pore volumes between 2 and 10 nm, significantly influenced MBP, with higher SSA values correlating with improved MBP and MBV.
6. The MBV of the tiles ranged between 5.68 and 7.94 g/m^2^ Δ%HR, which are the highest values reported for geopolymer structures in the literature.

In line with the findings from this study, these geopolymer tiles, with their superior moisture buffering and durability characteristics, offer significant potential in addressing various challenges in modern construction and building design. Below are examples of specific scenarios where these tiles could be effectively implemented:

1. Climate-controlled interiors: In areas with fluctuating indoor humidity levels, such as museums, archives, or healthcare facilities, geopolymer tiles could play a pivotal role in regulating moisture. By absorbing excess moisture during high humidity periods and releasing it when the environment becomes drier, these tiles contribute to a more stable, comfortable, and healthy indoor climate. This moisture regulation not only supports human well-being but also helps preserve delicate materials and documents.
2. Eco-friendly building projects: Geopolymer tiles could be integral to sustainable building practices. Their ability to naturally regulate indoor humidity can help reduce reliance on mechanical ventilation and air conditioning, thus lowering the overall energy consumption of a building. This would be particularly advantageous in regions with hot, humid climates where energy-efficient cooling is essential.
3. Moisture management in basement and underground spaces: Basements and underground structures are often prone to moisture accumulation and mold growth due to their proximity to groundwater. GP tiles, known for their superior moisture resistance and hygroscopic properties, can be used as flooring or wall cladding in these spaces. By controlling moisture levels, they can significantly reduce the risk of mold and enhance the long-term durability of these spaces.
4. Passive building designs in cold climates: In cold climates where moisture condensation can be a concern, GP tiles could be used to regulate humidity in buildings. By absorbing moisture from the air during condensation events and releasing it back when temperatures rise, these tiles contribute to energy-efficient buildings by reducing the need for constant ventilation. This could be particularly useful in residential homes, schools, or offices where maintaining a comfortable indoor air quality is crucial.

In conclusion, the hygroscopic GP tiles produced in this study demonstrated an exceptional moisture buffering performance and durability. Their ability to absorb and release moisture in response to environmental changes, combined with their overall strength and water resistance, positions them as a superior alternative to traditional building materials. These tiles can be implemented in various construction scenarios, addressing specific challenges related to moisture management, energy efficiency, and long-term building sustainability. The results of this study suggest that geopolymer tiles have significant potential to become a standard material for eco-friendly and resilient construction, particularly in applications where moisture control and durability are critical.

## Supporting Information Available

Tables of chemical compositions of five types of kaolin used in this study, the change in SSA values of MK750 kaolin at different heating rates and exposure times, climate chamber cycle data for five types of GP tile, tile absorption desorption data, and the calculated crystal size of kaolins using the Scherrer equation by XRD; figures of FTIR spectrum of the metakaolin powders, XRD graph of geopolymer tiles, SEM-EDS images of geopolymer tiles, particle size distribution analyses of the four different kaolins, detailed XRD spectra of kaolins, and three-point bending strength device (PDF)
