# Supplementary material for: Innovative Geopolymer Tiles for Indoor Humidity Control: A Comparative Study of Moisture Buffering Performance
Source: ACS Omega. 2025 Mar 3;10(9):9197–209. doi: 10.1021/acsomega.4c09422 (PMC11904677; doi:10.1021/acsomega.4c09422)
Supplement: Supplementary file 1 — ao4c09422_si_001.pdf [file ao4c09422_si_001.pdf]

Innovative Geopolymer Tiles for Indoor Humidity Control: A Comparative Study of Moisture Buffering Performance

*Gürkan Akarken[a,b], Yildiz Yildirim[c], Ugur Cengiz[b,d]**

[a]*Department of Energy Resources and Management, Faculty of Engineering, Çanakkale Onsekiz Mart University, Çanakkale, Türkiye*

[b]*AFC Green Technologies R&D, Canakkale Technopark, Sarıcaeli, 17100, Çanakkale, Turkiye*

[c]*Kale Ceramic R&D Department, Canakkale, 17400, Türkiye*

[d]*Surface Science Research Laboratory, Department of Chemical Engineering, Faculty of Engineering, Çanakkale Onsekiz Mart University, Çanakkale, Türkiye*

*Corresponding author: ucengiz@comu.edu.tr

Phone: +90 (286) 218 00 18

Supporting Information (SI)

SI Table S1. Chemical composition (wt %) of five types of kaolin and L05 metakaolin

**Oxides**	**MK750***	**L05****	**K2**	**CC31**	**MBA**	**CAMP S4**
SiO_2	53.0	51.23	47.29	50.09	48.53	56.58
Al_2O_3	43.8	41.50	36.55	35.32	35.98	28.60
TIO_2	1.70	1.39	0.32	0.44	0.13	0.29
Fe_2O_3	0.43	0.94	0.88	0.78	0.88	1.62
CaO	0.02	0.68	0.65	0.12	0.05	0.30
MgO	0.03	0.63	0.10	0.14	0.31	0.84
Na_2O	0.23	0.34	0.07	0.10	0.10	0.48
K_20	0.19	0.47	0.96	0.58	2.39	2.50
L.O.I.***	0.46	2.54	12.68	12.34	11.47	8.28

***** Commercial reference kaolin ** Commercial reference metakaolin *** Lost on ignition

SI Table S2. The change in SSA values of MK750 kaolin at different heating rates (°C/min) and exposure times (h).

**Exposure Time (hours)**	**SSA values (m^2/g)**	
	**20 °C/min**	**50 °C/min**
2	18.20	19.74
4	17.11	18.88
6	15.42	17.86
8	13.50	16.05
10	10.00	13.75

SI Table S3. L05 geopolymer tile's press pressure – strength change values

**Press Pressure (kg/cm^2)**	**Strength (kg/cm^2 *)**
250	26.2 ± 0.2
300	31.3 ± 0.1
350	40.1 ± 0.2
400	49.8 ± 0.2

* Strength values are the average of three 5x10 cm tablets

SI Table S4. Climate chamber cycle datas for five types of GP tile

**Geopolymer**		**a (mm)**	**h(mm)**	**Volume (cm^3)** $3\sqrt{3}.a^2.h/2$
	1	30.19	8.13	19.25
	2	30.15	8.32	19.65
**L05**	3	30.12	8.18	19.28
	4	30.18	8.22	19.45
	5	30.16	7.67	18.13
	1	29.68	8.02	18.35
	2	29.79	8.05	18.56
**K2**	3	29.94	8.30	19.33
	4	29.89	8.24	19.13
	5	29.83	8.12	18.77
	1	29.74	8.32	19.12
	2	29.80	7.83	18.07
**CC31**	3	29.87	8.21	19.03
	4	29.53	7.51	17.01
	5	29.74	7.85	18.04
	1	30.08	8.12	19.09
	2	30.15	8.05	19.01
**MBA**	3	30.24	8.30	19.72
	4	29.98	8.24	19.24
	5	30.00	8.12	18.99
	1	29.85	8.00	18.52
	2	29.79	7.98	18.40
**CAMPS S4**	3	30.05	7.86	18.44
	4	29.62	8.01	18.26
	5	30.41	7.75	18.62

SI Table S5. L05 tile absorption desorption datas*

**Volume (cm^3)**		**19.25**	**19.65**	**19.28**	**19.45**	**18.13**
Ads. Moisture(g)		0.9788	0.9863	0.9548	0.9958	0.9561
g/cm^3		0.051	0.050	0.050	0.051	0.053
**AVR**		**0.0509**				
Dsb. Moisture (g)		0.9482	0.9337	0.9648	0.9472	0.9208
g/cm^3	**1. Cycle**	0.049	0.048	0.050	0.049	0.051
**AVR**		**0.0493**				
Adsorb Moisture (g)		0.9461	0.9758	0.9415	0.9617	0.9114
g/cm^3		0.049	0.050	0.049	0.049	0.050
**AVR**		**0.0495**				
Dsb. Moisture (g)		0.9049	0.911	0.9166	0.8997	0.9000
g/cm^3	**2. Cycle**	0.047	0.046	0.048	0.046	0.050
**AVR**		**0.0474**				
Ads. Moisture (g)		0.8991	0.9051	0.8881	0.9167	0.8869
g/cm^3		0.047	0.046	0.046	0.047	0.049
**AVR**		**0.0470**				
Dsb. Moisture (g)		0.8098	0.8215	0.8367	0.8196	0.8035
g/cm^3	**3. Cycle**	0.042	0.042	0.043	0.042	0.044
**AVR**		**0.0427**				

*Measurements were taken 24 hours of interval

SI Table S6. K-2 tile absorption desorption datas*

**Volume (cm^3)**		**18.35**	**18.56**	**19.33**	**19.13**	**18.77**
Ads. Moisture (g)		0.9196	0.9165	0.9362	0.9316	0.9543
g/cm3		0.050	0.049	0.048	0.049	0.051
**AVR**		**0.0495**				
Dsb. Moisture (g)	**1. Cycle**	0.8588	0.8337	0.8698	0.8489	0.8808

g/cm3		0.047	0.045	0.045	0.044	0.047
**AVR**		**0.0456**				
Adsb. Moisture (g)		0.8560	0.8465	0.8843	0.8517	0.8766
g/cm3		0.047	0.046	0.046	0.045	0.047
**AVR**		**0.0458**				
Dsb. Moisture (g)	**2. Cycle**	0.8094	0.8174	0.8093	0.7923	0.8167
g/cm3		0.044	0.044	0.042	0.041	0.044
**AVR**		**0.0430**				
Ads. Moisture (g)		0.8370	0.8294	0.8268	0.8188	0.8236
g/cm3		0.046	0.045	0.043	0.043	0.044
**AVR**		**0.0440**				
Dsb. Moisture (g)	**3. Cycle**	0.7998	0.8007	0.8216	0.7852	0.7819
g/cm3		0.044	0.043	0.043	0.041	0.042
**AVR**		**0.0424**				

*Measurements were taken 24 hours of interval

SI Table S7. CC-31 tile absorption desorption datas*

**Volume (cm^3)**		**19.12**	**18.07**	**19.03**	**17.01**	**18.04**
Ads. Moisture (g)	**1. Cycle**	0.8929	0.8512	0.8745	0.8468	0.8519
g/cm3		0.047	0.047	0.046	0.050	0.047
**AVR**		**0.0474**				
Dsb. Moisture (g)		0.8746	0.889	0.8563	0.8715	0.8173
g/cm3		0.046	0.049	0.045	0.051	0.045
**AVR**		**0.0473**				
Ads. Moisture (g)	**2. Cycle**	0.8699	0.8857	0.8544	0.8705	0.8149
g/cm3		0.045	0.049	0.045	0.051	0.045

**AVR**		**0.0472**				
Dsb. Moisture (g)		0.8806	0.8652	0.8377	0.8536	0.8054
g/cm3		0.046	0.048	0.044	0.050	0.045
**AVR**		**0.0466**				
Ads. Moisture (g)		0.8465	0.8405	0.8068	0.8241	0.7782
g/cm3		0.044	0.047	0.042	0.048	0.043
**AVR**		**0.0450**				
Dsb. Moisture (g)		0.8314	0.8192	0.8254	0.801	0.8017
g/cm3	**3. Cycle**	0.043	0.045	0.043	0.047	0.044
**AVR**		**0.0447**				

*Measurements were taken 24 hours of interval

SI Table S8. MB-A tile absorption desorption datas*

**Volume (cm^3)**		**19.09**	**19.01**	**19.72**	**19.24**	**18.99**
Ads. Moisture (g)		0.7765	0.7769	0.7991	0.8014	0.7888
g/cm3		0.041	0.041	0.041	0.042	0.042
**AVR**		**0.0411**				
Dsb. Moisture (g)		0.7746	0.788	0.7612	0.7782	0.7891
g/cm3		0.041	0.041	0.039	0.040	0.042
**AVR**	**1. Cycle**	**0.0405**				
Adsorb Nem(g)		0.7699	0.7841	0.7844	0.7815	0.8019
g/cm3	**2. Cycle**	0.040	0.041	0.040	0.041	0.042

**AVR**		**0.0408**				
Dsb Moisture (g)		0.7708	0.7691	0.782	0.7736	0.7725
g/cm3		0.040	0.040	0.040	0.040	0.041
**AVR**		**0.0403**				
Ads. Moisture (g)		0.7801	0.7701	0.7966	0.7741	0.7819
g/cm3		0.041	0.041	0.040	0.040	0.041
**AVR**		**0.0406**				
Dsb. Moisture (g)		0.7355	0.7461	0.7854	0.7811	0.7717
g/cm3	**3. Cycle**	0.039	0.039	0.040	0.041	0.041
**AVR**		**0.0398**				

*Measurements were taken 24 hours of interval

SI Table S9. CAMP S-4 tile absorption desorption datas*

**Volume (cm^3)**		**18.52**	**18.4**	**18.44**	**18.26**	**18.62**
Ads. Moisture (g)		0.676	0.6566	0.6994	0.6557	0.6987
g/cm3		0.037	0.036	0.038	0.036	0.038
**AVR**		**0.0367**				
Dsb. Moisture (g)		0.6334	0.6954	0.6991	0.6793	0.6465
g/cm3	**1. Cycle**	0.034	0.038	0.038	0.037	0.035
**AVR**		**0.0364**				
Ads. Moisture (g)		0.6513	0.6331	0.6319	0.6184	0.697
g/cm3		0.035	0.034	0.034	0.034	0.037
**AVR**	**2. Cycle**	**0.0350**				
Dsb. Moisture (g)		0.6115	0.6172	0.6089	0.6186	0.6174
g/cm3		0.033	0.034	0.033	0.034	0.033

**AVR**		**0.0333**				
Ads. Moisture (g)		0.6485	0.6543	0.6481	0.6388	0.6194
g/cm3		0.035	0.036	0.035	0.035	0.033
**AVR**		**0.0348**				
Dsb. Moisture (g)		0.6011	0.5971	0.5976	0.5586	0.576
g/cm3	**3. Cycle**	0.032	0.032	0.032	0.031	0.031
**AVR**		**0.0318**				

*Measurements were taken 24 hours of interval

SI Table S10: The calculated crystal size of kaolins using the Scherrer Equation by XRD (°A)

	Calculated Crystal Size			
	**Peak 7.14°A**	**Peak 3.57°A**	**Peak 2.35°A**	**AVR**
**K-2**	791	677	739	736
**CC-31**	1595	1340	1608	1514
**MB-A**	2393	2086	2585	2355
**CAMP S-4**	1126	940	1030	1032

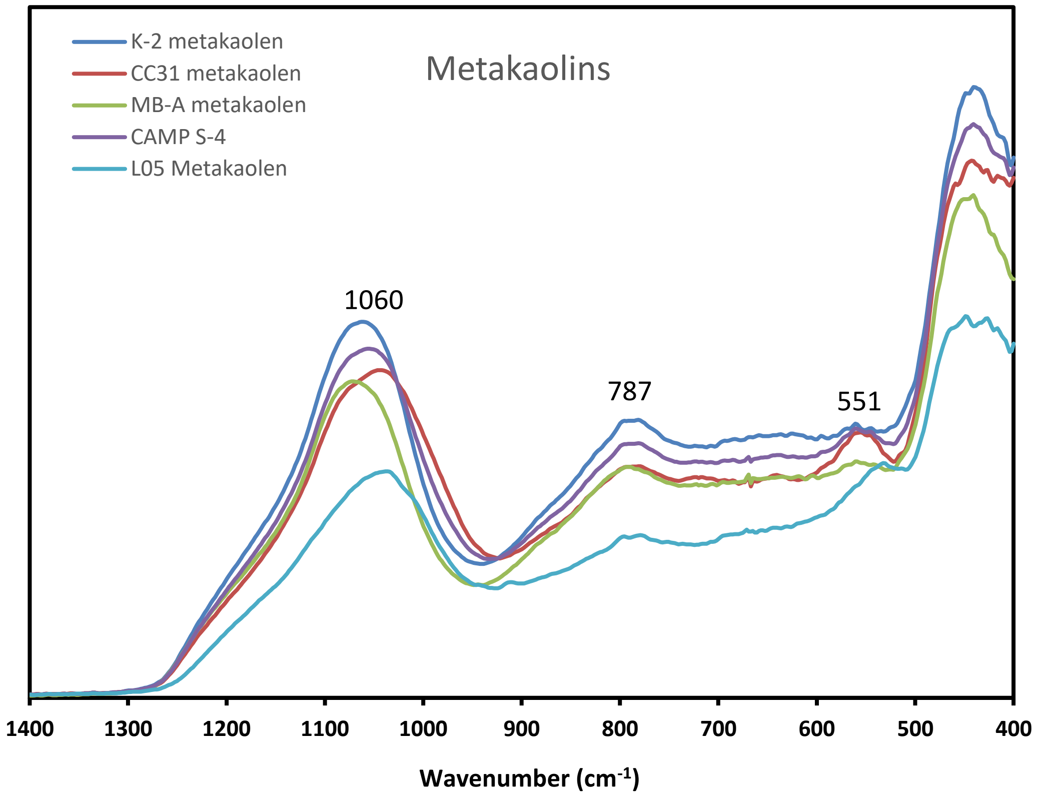

SI Fig. S1. FTIR spectrum of the metakaolin powders

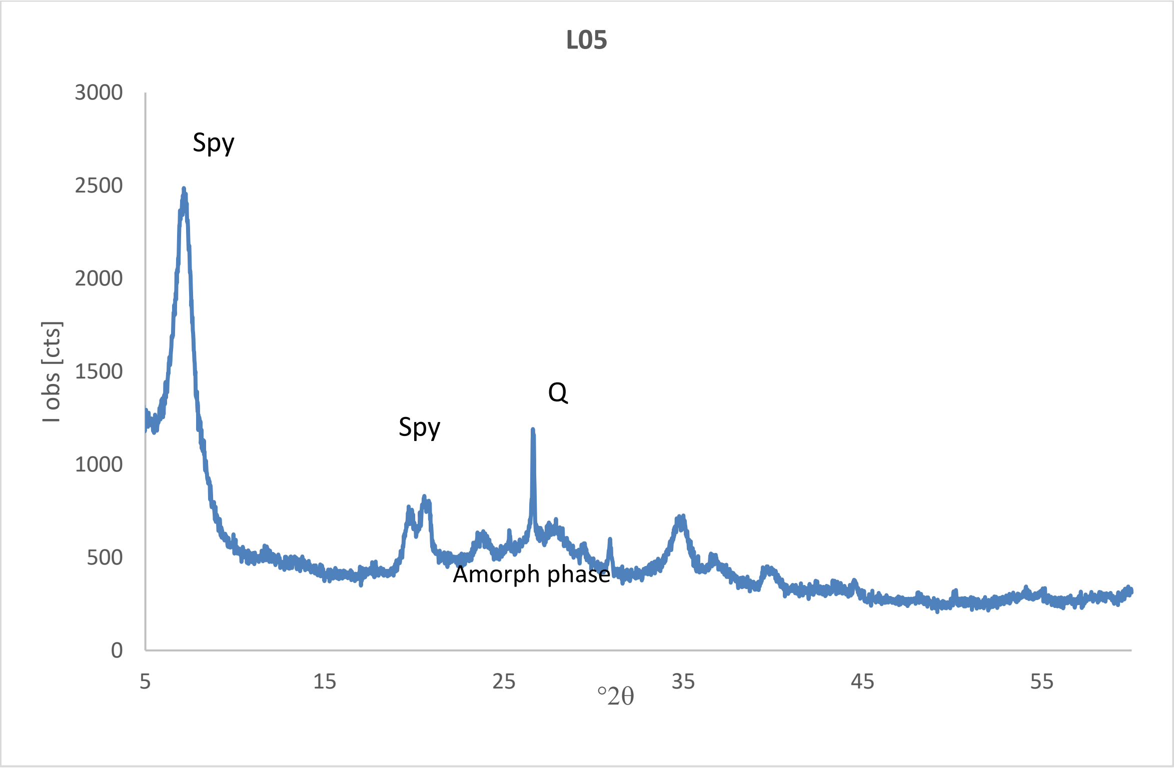

SI Fig. S2. XRD graph of geopolymer tile prepared with L05 Metakaolin

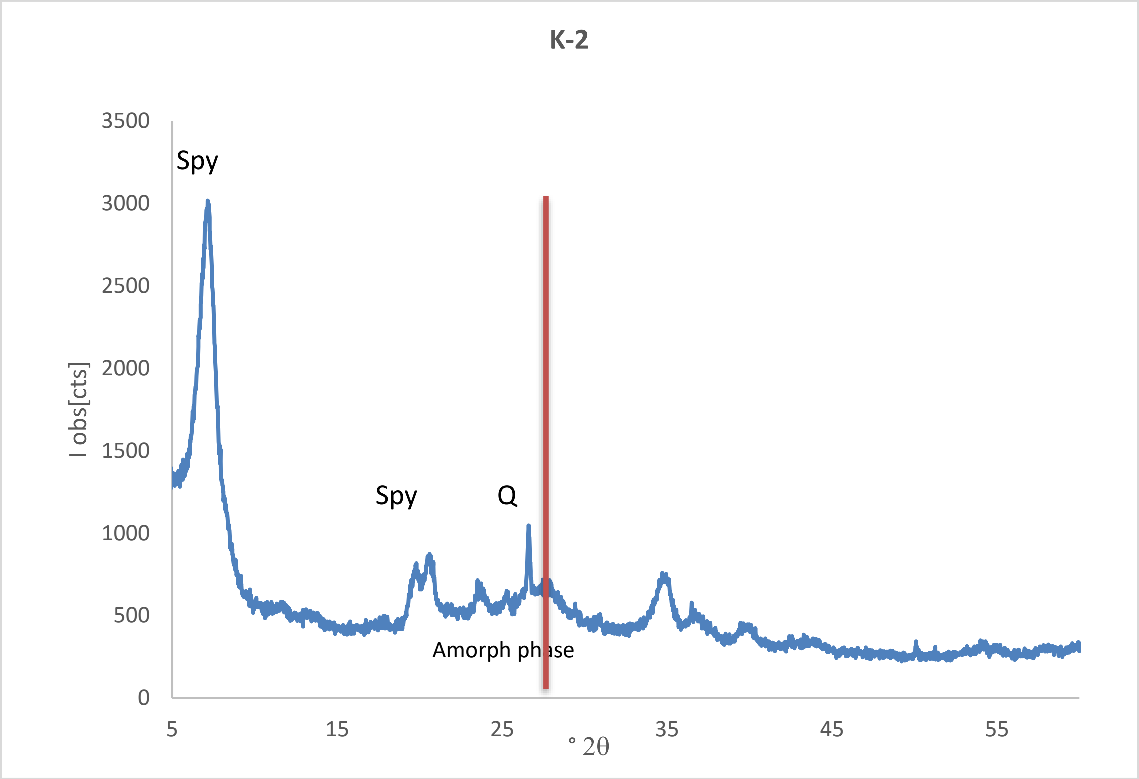

SI Fig. S3. XRD graph of geopolymer tile prepared with K2 Metakaolin

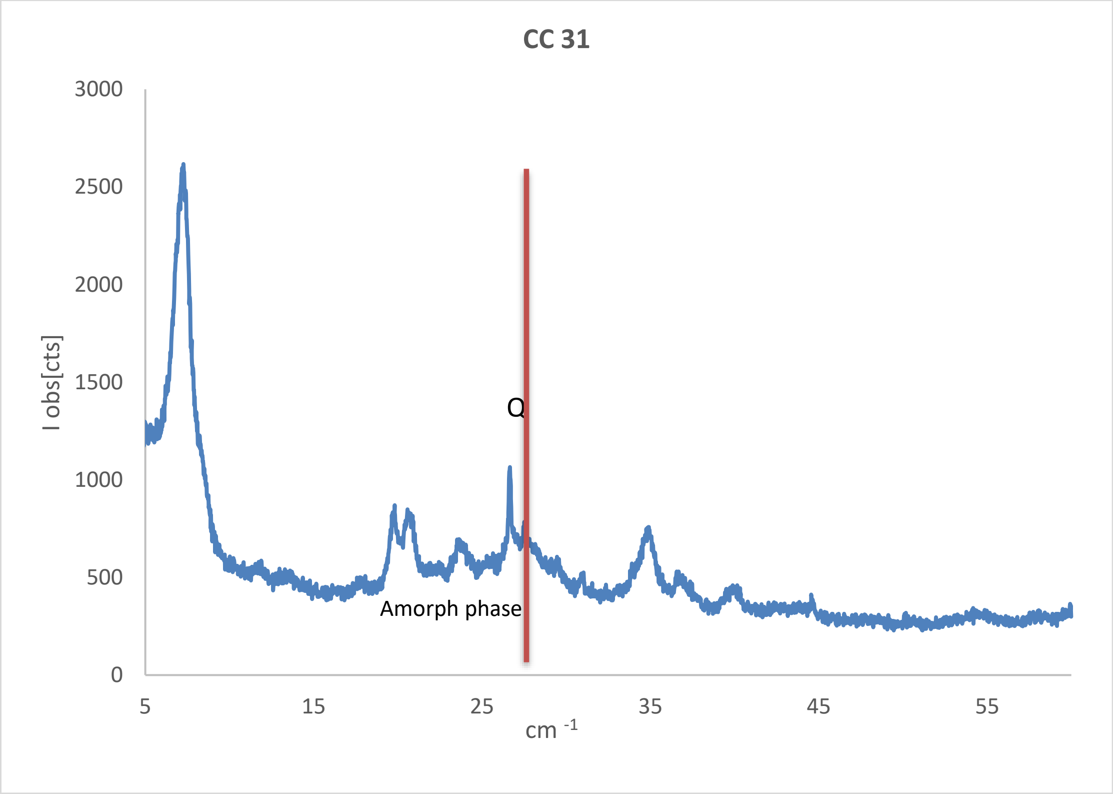

SI Fig. S4. XRD graph of geopolymer tile prepared with CC31 Metakaolin

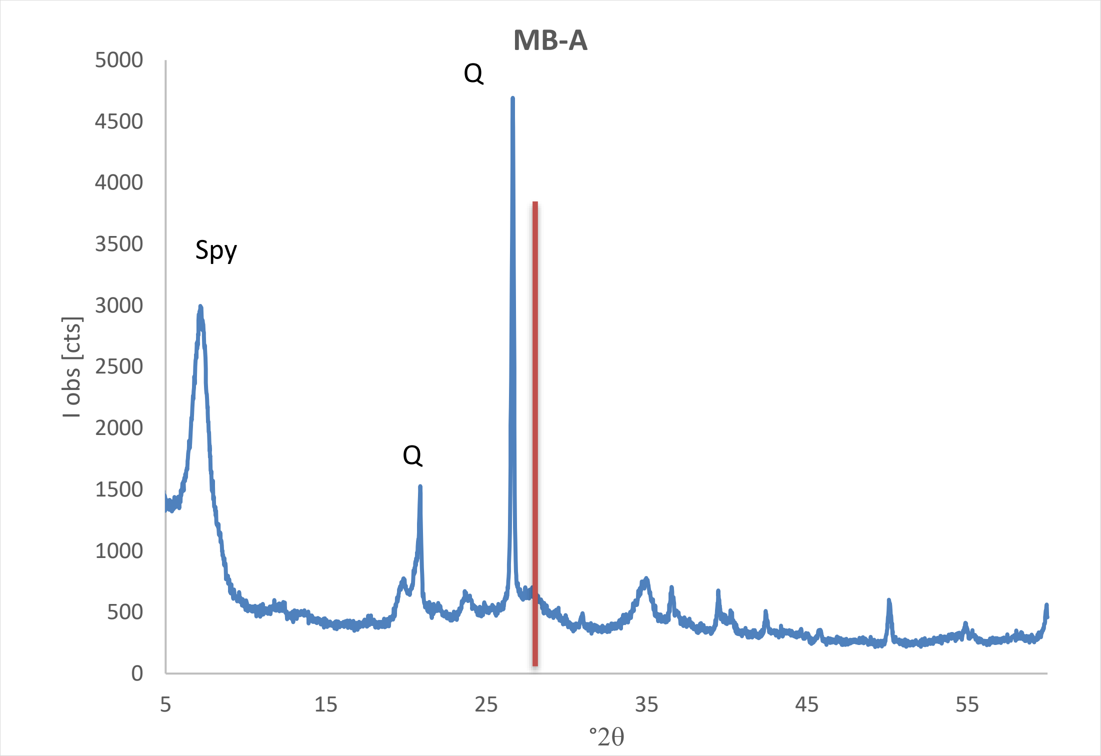

SI Fig. S5. XRD graph of geopolymer tile prepared with MBA Metakaolin

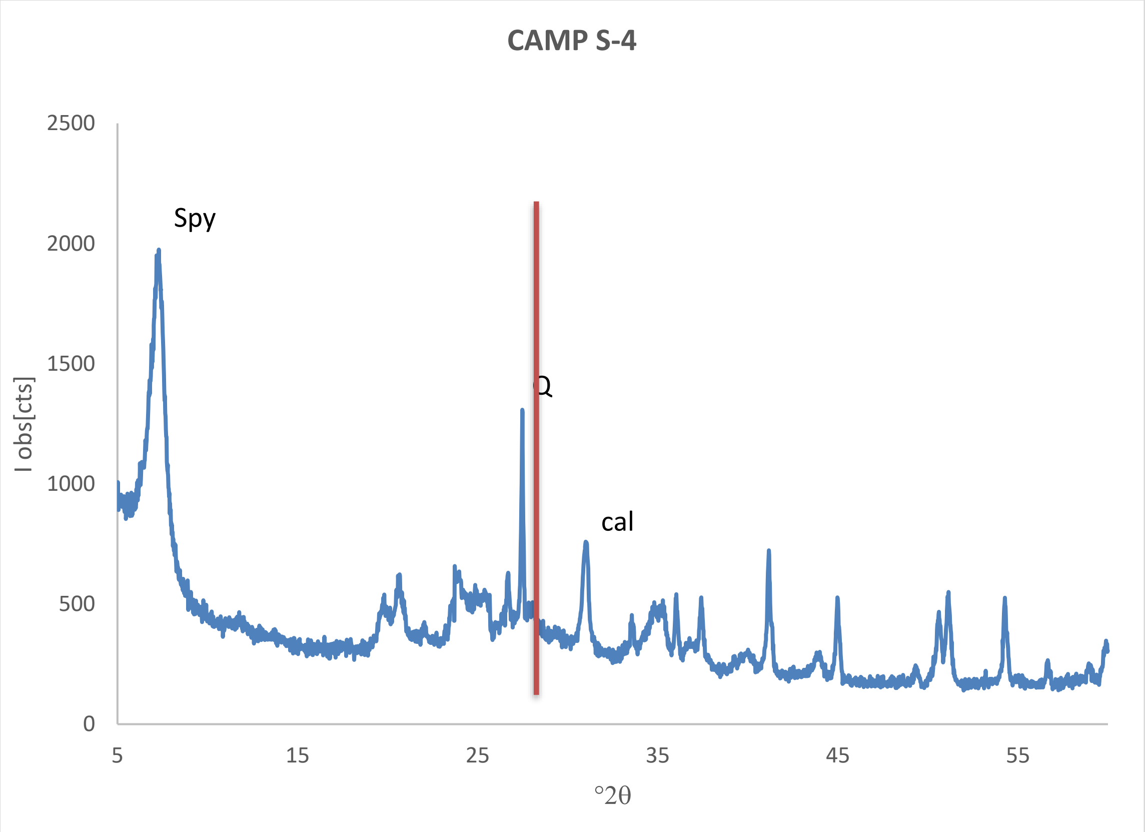

SI Fig. S6. XRD graph of geopolymer tile prepared with CAMP S4 Metakaolin

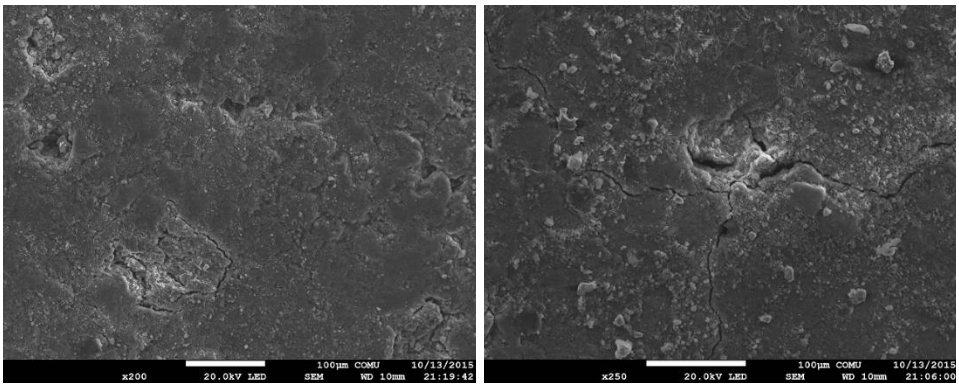

SI Fig. S7 SEM images of L05 geopolymer tiles - 100µm

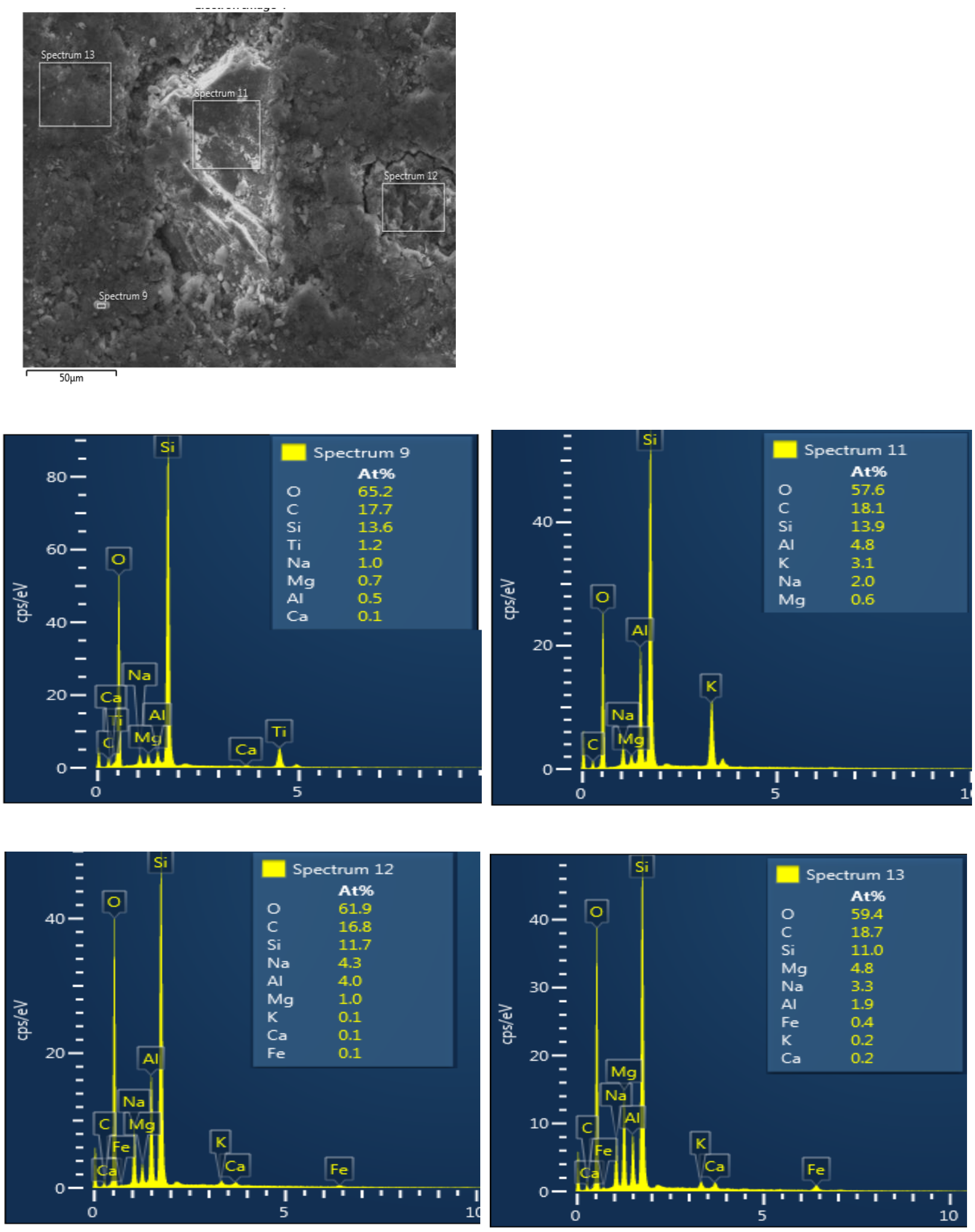

SI Fig. S8 SEM, EDS images of L05 geopolymer tiles -50µm

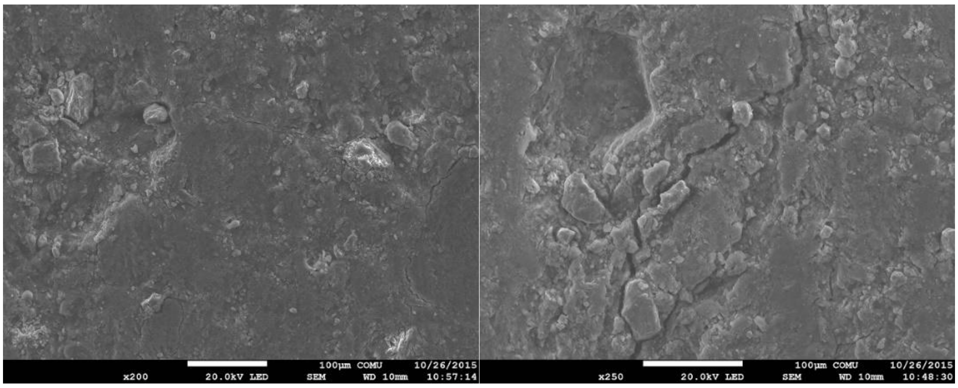

SI Fig. S9 SEM images of K2 geopolymer tiles - 100µm

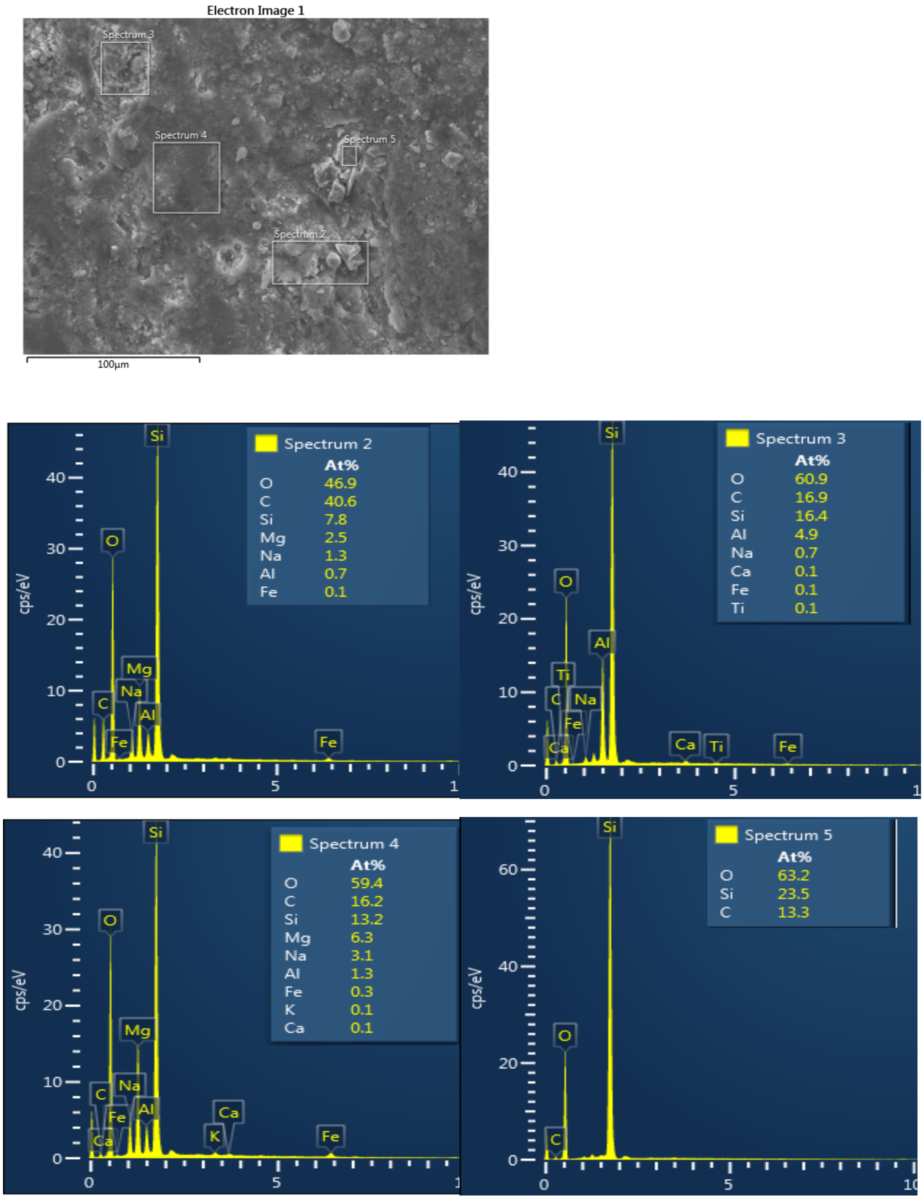

SI Fig. S10 SEM-EDS images of K-2 geopolymer tiles -100 µm

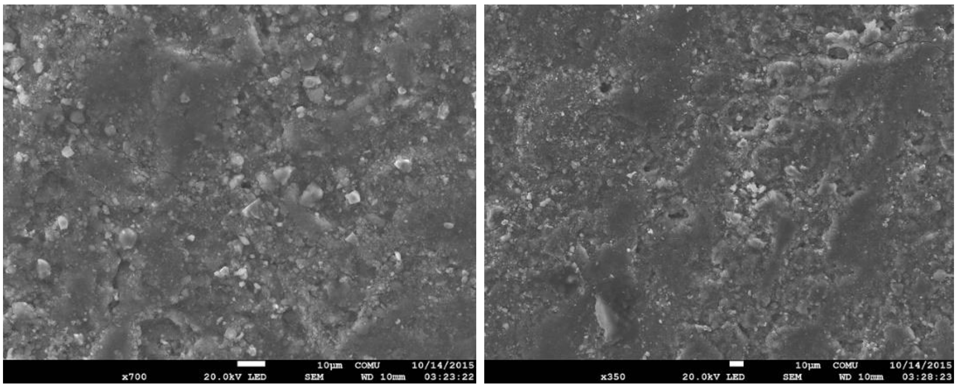

SI Fig. S11 SEM images of CC31 geopolymer tiles -10µm

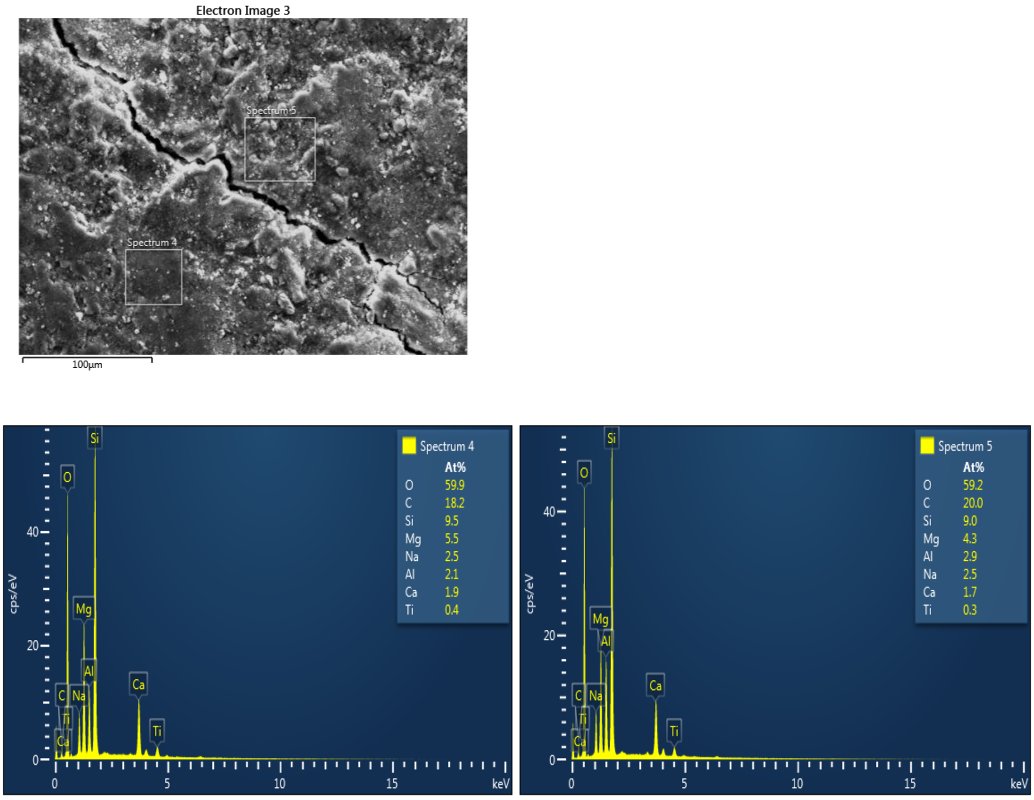

SI Fig. S12 SEM-EDS images of CC31 geopolymer tiles -100 µm

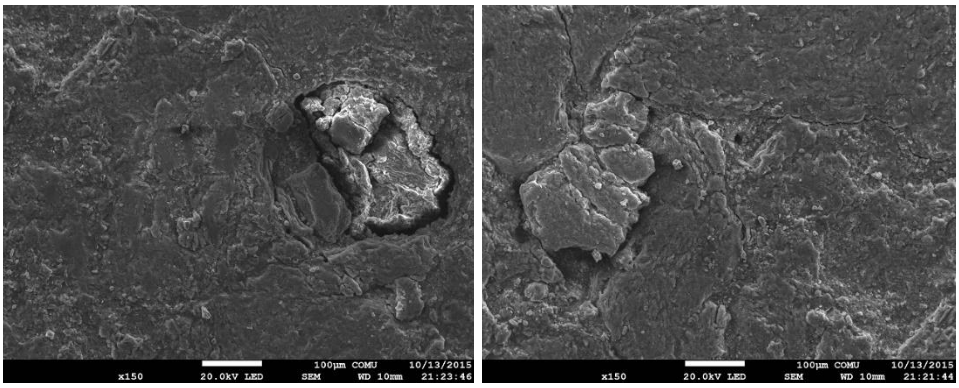

SI Fig. S13 SEM images of MBA geopolymer tiles -100µm

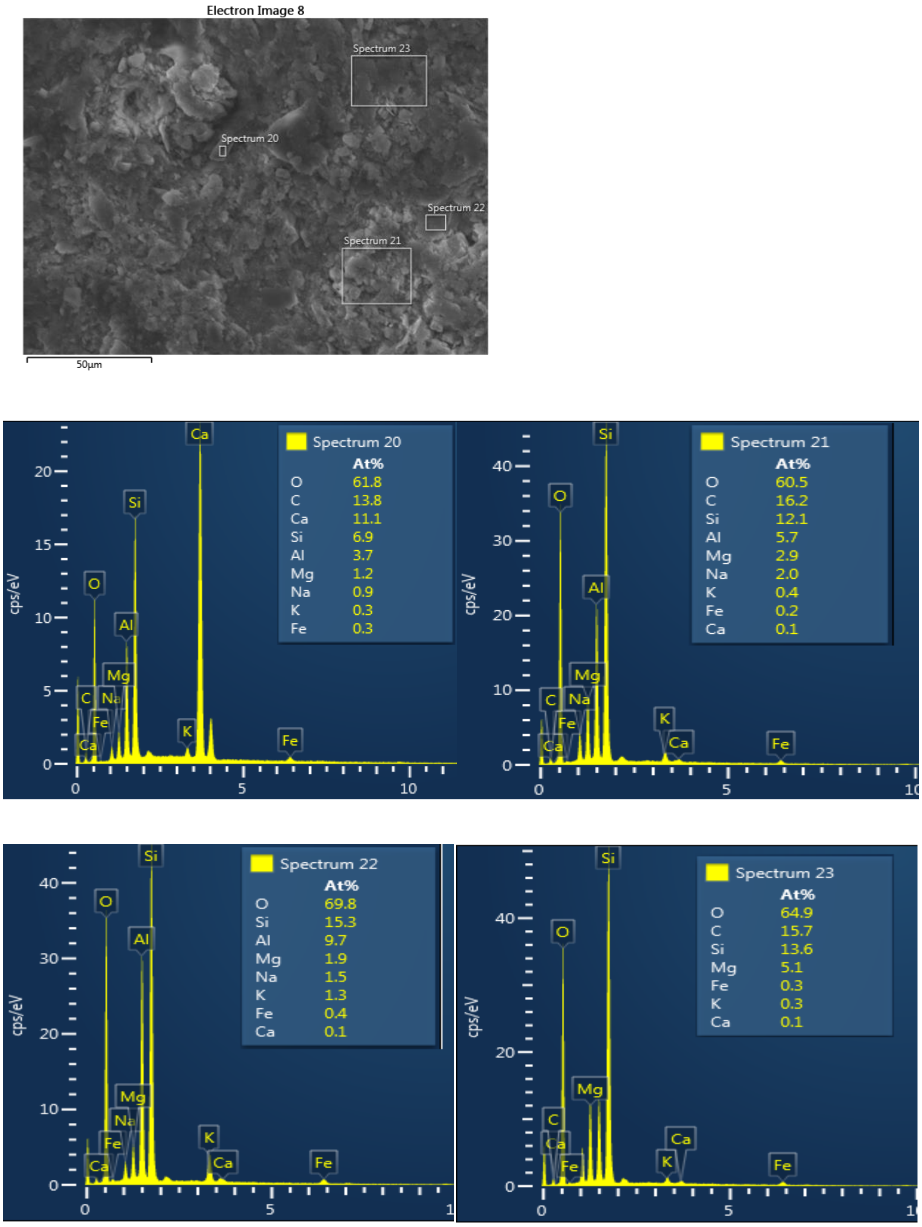

SI Fig. S14 SEM-EDS images of MBA geopolymer tiles - 50µm

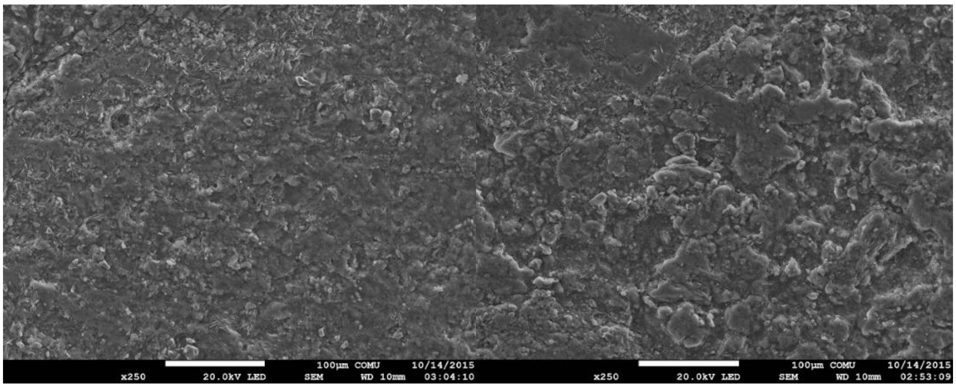

SI Fig. S15 SEM images of CAMPS S4 geopolymer tile -100µm

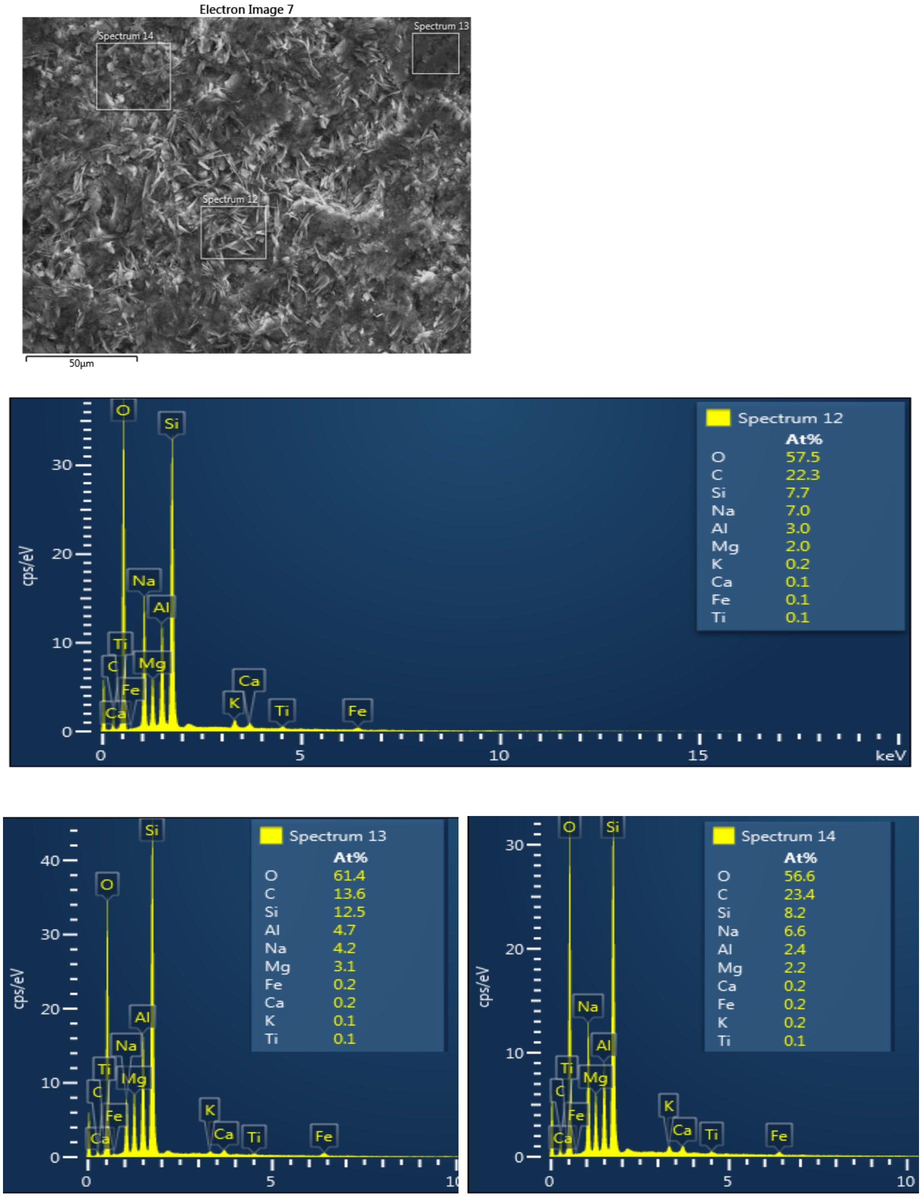

SI Fig. S16 SEM-EDS images of CAMPS S4 geopolymer tiles -50µm

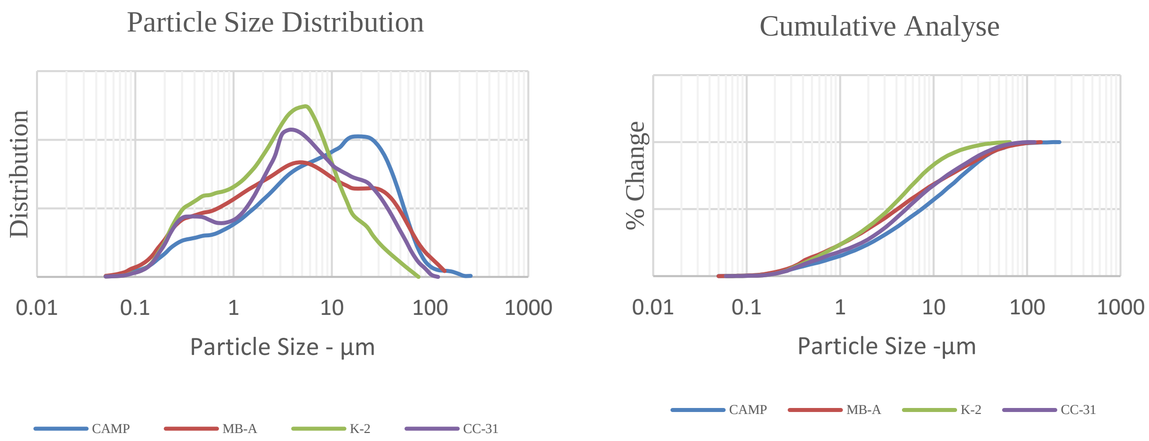

SI Fig. S17. Particle size distribution analyses of the four different kaolins
